# Delivering Opportunistic Behavior Change Interventions: a Systematic Review of Systematic Reviews

**DOI:** 10.1007/s11121-020-01087-6

**Published:** 2020-02-17

**Authors:** Chris Keyworth, Tracy Epton, Joanna Goldthorpe, Rachel Calam, Christopher J. Armitage

**Affiliations:** 1 Manchester Centre for Health Psychology, Division of Psychology and Mental Health, School of Health Sciences, Faculty of Biology, Medicine and Health, The University of Manchester, Coupland 1 Building–Room G3, Oxford Road, Manchester, M13 9PL UK; 2 Manchester University NHS Foundation Trust, Manchester Academic Health Science Centre, M13 9PL, Manchester, UK

**Keywords:** Prevention, Health professional-patient interaction, Communication, Health behavior, Systematic review

## Abstract

Opportunities for healthcare professionals to deliver health behavior change interventions are often missed, but understanding the barriers and enablers to this activity is limited by a focus on defined specialisms/health conditions. This systematic review of systematic reviews collates all the evidence across professional groups to provide guidance to policy makers for implementing healthcare professional delivery of behavior change interventions. Eight electronic databases were searched for systematic reviews reporting patient-facing healthcare professionals’ (e.g., general practitioners, nurses) barriers and enablers to delivering behavior change interventions (diet, physical activity, alcohol reduction, smoking cessation, and weight management). A narrative synthesis was conducted. Thirty-six systematic reviews were included. Four themes emerged as both barriers and enablers: (1) perceptions of the knowledge or skills needed to support behavior change with patients, (2) perceptions of the healthcare professional role, (3) beliefs about resources and support needed, and (4) healthcare professionals’ own health behavior. There were four cross-disciplinary barriers: (1) perceived lack of time, (2) perceived lack of prioritization of health behavior change, (3) negative attitudes towards patients and perceptions of patient risk, and (4) perceptions of patient motivation. The three enablers were as follows: (1) training, (2) context, and (3) attitudes towards delivering interventions. To enhance healthcare professionals’ delivery of behavior change interventions, policy makers should (a) address perceptions about patient need for interventions, (b) support diverse professional groups to identify opportunities to deliver interventions, and (c) encourage professionals to focus on prevention and management of health conditions.

**Electronic supplementary material:**

The online version of this article (10.1007/s11121-020-01087-6) contains supplementary material, which is available to authorized users.

## Introduction

Unhealthy behaviors are important risk factors for long-term conditions such as cardiovascular diseases, diabetes, and cancer, making health behavior change a medical issue of worldwide importance (World Health Organization 2017). Public health strategies are used internationally to compel healthcare professionals to deliver opportunistic behavior change interventions to patients (Public Health England 2016; The Royal Australian College of General Practitioners (RACGP) (2017)), including smoking cessation, improving diet, increasing physical activity, and reducing alcohol intake. Healthcare professionals are an expected and trusted source of health behavior change advice (McPhail and Schippers 2012), and patients often welcome advice about behavior change even during routine primary care consultations, where health behavior is not the primary focus (Aveyard et al. 2012).

Behavior change interventions can be delivered in as few as 30 s (Aveyard et al. 2016), and are cost effective, with the cost of delivery falling below the cost per quality-adjusted life-year (QALY) thresholds (National Institute for Health and Care Excellence 2014). Although interventions delivered by patient-facing healthcare professionals enable interventions to have maximum reach, a lack of time to deliver interventions (Heslehurst et al. 2014), or a perceived lack of knowledge or skills to deliver behavior change interventions (Yousefzadeh et al. 2016) may impede delivery.

Although there is a growing knowledge base in relation to the barriers and enablers to healthcare professionals’ implementation of behavior change interventions as part of routine practice, there are two important limitations of existing systematic reviews. First, previous systematic reviews focus specifically on defined healthcare professionals in the context of managing specific conditions, or healthcare professionals working within specific contexts such as primary or secondary care settings. For example, previous systematic reviews have focused on weight management interventions delivered by midwives during pregnancy (Heslehurst et al. 2014), smoking cessation interventions delivered by anesthesiologists (Yousefzadeh et al. 2016), physical activity interventions delivered by nurses as part of cancer care (Webb et al. 2016), or health behavior change support by dentists (Lala et al. 2017). A second limitation of existing systematic reviews is the lack of focus on common barriers and enablers that are shared across professional groups. For example, while the literature suggests barriers to delivering interventions include a perceived lack of time in the consultation, these are reported in the context of specific disciplines such as nursing (van Dillen and Hiddink 2014), general practice (Stead et al. 2009), midwifery (Heslehurst et al. 2014), and anesthesiology (Yousefzadeh et al. 2016). Given the conflicting priorities and remits of healthcare professionals working in different specialisms, it is often difficult to extend the findings of previous reviews to other healthcare professionals working in different specialisms. Subsequently, there is currently no understanding of any shared barriers or enablers to delivering behavior change interventions across diverse healthcare professional groups with conflicting priorities.

It is important to understand and synthesize the cross-disciplinary barriers and enablers to delivering behavior change interventions as this offers the opportunity to (1) design and deliver generic professional practice interventions across diverse professional groups to support healthcare professionals to deliver interventions, targeting shared barriers and enablers; and (2) facilitate the implementation of public health policies designed for all healthcare professionals to deliver behavior change interventions at scale.

The number of systematic reviews examining healthcare professionals’ delivery of behavior change interventions is increasing (a PubMed search found two reviews in the 1970s, 15 reviews in the 1980s, 49 reviews in the 1990s, 225 reviews in the 2000s, and 702 reviews in the 2010s). Systematic reviews of systematic reviews have been used more recently as a way of providing a broader overview of a particular field, compared with systematic reviews that are conducted within a specific discipline (French et al. 2017). No systematic review to date has aimed to collate all of the available evidence in relation to the common barriers and enablers to healthcare professionals delivering behavior change interventions. A recent national survey showed that healthcare professionals deliver behavior change interventions in just 50% of cases where they thought patients would benefit from interventions (Keyworth et al. 2018). The specific reasons as to why this is the case, particularly across diverse specialisms, is currently unclear. Therefore, the primary aim of the present systematic review of systematic reviews was to provide a broad overview of the shared barriers and enablers of delivering behavior change interventions (in relation to diet, physical activity, alcohol reduction, smoking cessation, and weight management) across diverse healthcare professional groups.

## Method

The present review was registered in PROSPERO (42017059888).

### Inclusion Criteria

Reviews were included if they were (a) systematic (i.e., provided details of a systematic search strategy such as a database list, search terms, inclusion and exclusion criteria); (b) reported patient-facing healthcare professionals’ (e.g., GPs, nurses, midwives) barriers to and facilitators of providing healthy lifestyle advice (i.e., diet, physical activity, alcohol reduction, smoking cessation, and weight management—including obesity); and (c) were in English. There were no restrictions on type of health condition presented in the review (e.g., heart disease, diabetes). Reviews were included that contained both qualitative (e.g., semi-structured interviews) and quantitative studies (e.g., questionnaires and surveys).

### Exclusion Criteria

Reviews were excluded if they (a) did not report healthcare professionals’ barriers and facilitators of providing health behavior change interventions, (b) were unsystematic (i.e., did not provide details of a systematic search strategy such as a database list, search terms, inclusion and exclusion criteria), (c) were not of studies conducted with patient-facing healthcare professionals, and (d) were in a language other than English.

### Primary Outcome

The barriers and facilitators to healthcare professionals providing health behavior change advice and/or interventions to patients.

### Search Strategy

Eight electronic databases were searched (PubMed, Web of Science, CINAHL, PsycINFO, Embase, Cochrane, SPORTDiscus, Scopus) from inception to November 2018 using key MeSH terms. A hand search of the reference lists of included systematic reviews was also conducted. The search strategies are presented in full in Supplementary File A. Titles and abstracts were independently screened by two authors resulting in 92.60% agreement (1814/1959). Articles meeting the inclusion criteria were subject to full text review independently by three authors, with 67.90% agreement (74/109). Disagreements were discussed and resolved with the full team.

### Quality Assessment

Full text systematic reviews were assessed for quality using the AMSTAR measurement tool, developed to assess methodological quality of systematic reviews (Shea et al. 2009).

### Data Extraction/Coding of Primary Systematic Reviews

The key information was extracted by a single reviewer using a standardized data extraction tool, which was pilot-tested with a small sample of systematic reviews, and subsequently refined where necessary by the research team. Information included review information (e.g., date, inclusion/exclusion criteria, dates covered, study quality), study characteristics (e.g., study design, type of behavior examined, type of healthcare professional), sample characteristics where available (e.g., age, ethnicity, nationality, gender), the barriers and facilitators reported in each review (extracted verbatim from the results and discussion sections of each systematic review), other study characteristics (e.g., date of publication, publication status, quality of studies), review characteristics (e.g., overlap of studies), and author conclusions.

### Data Synthesis

A narrative synthesis, combining the results of reviews reporting (a) qualitative studies, (b) quantitative, and (c) both qualitative and quantitative studies was conducted to identify the reported barriers and facilitators to healthcare professionals providing health behavior change advice to patients. Extracted information about the barriers and enablers to healthcare professionals delivering health behavior change advice and/or interventions was exported to NVivo version 11, which was used to organize and manage the key findings. To establish trustworthiness in the findings of the analysis, discussions among the entire study team were held at monthly intervals to develop the coding framework, and to discuss, refine, and group the emerging codes into overall themes. All study authors were involved in establishing the conceptual framework.

## Results

**Fig. 1:**
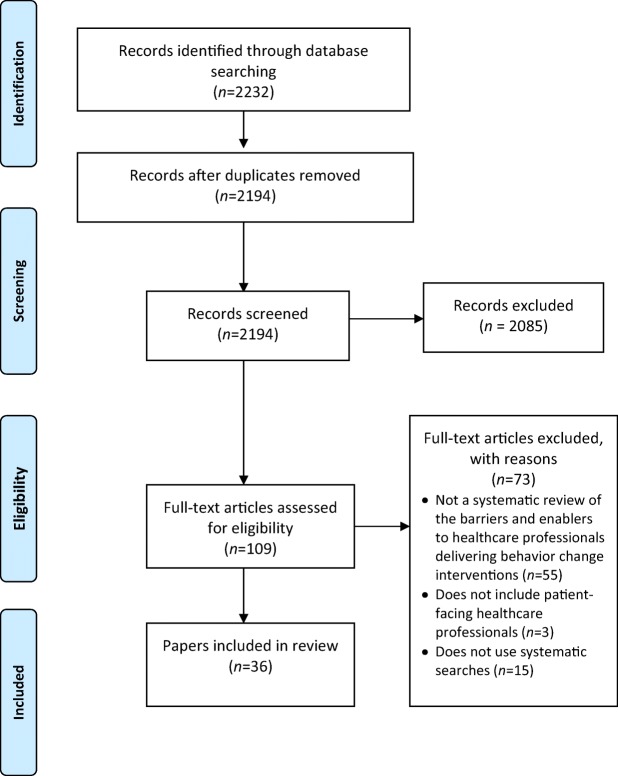
Flow diagram of papers included in the systematic review of systematic reviews

The literature search identified 2194 references after duplicates were removed. One hundred and nine references were selected and the full texts checked against the inclusion criteria. This resulted in thirty-six reviews (the full list of included reviews is presented in Supplementary File B). The main reasons for excluding reviews at the final stage were as follows: they did not review the barriers and enablers to healthcare professionals delivering behavior change interventions (*N* = 55), did not include patient-facing healthcare professionals (*N* = 3), or did not use systematic searches (*N* = 15; see Fig. 1).

### Quality of Included Reviews

The quality of the thirty-six included systematic reviews varied considerably according to the AMSTAR assessment tool. There was one meta-analysis, which scored 7/11. All other systematic reviews (without meta-analysis; *n* = 35) scored between 0/9 and 7/9, with a mean score of 3.8/7. Of the 35 systematic reviews that did not include meta-analyses, one scored 7/9.

### Overlap Between Reviews

The number of studies included in each systematic review varied from 4 studies (Verhaeghe et al. 2011) to 102 studies (Oxman et al. 1995), with a median of twenty studies. There were 958 unique studies included across all systematic reviews. Overlap of studies across the reviews ranged from 0 to 96%, with a mean of 16.92%. Thirteen studies (36.1%) had no overlap and appeared in only one systematic review.

### Systematic Review Characteristics

As shown in Supplementary File C (review characteristics), of the 36 systematic reviews, 4 (11%) are reviews of qualitative studies (interviews, focus groups), 13 (35%) are reviews reporting quantitative studies (trials, observational studies, self-report surveys), 18 (50%) are systematic reviews including both qualitative and quantitative studies, and 1 review (3%) did not state their inclusion criteria. One systematic review was conducted in the 1990s, seven systematic reviews were conducted in the 2000s, and 28 systematic reviews were conducted in the 2010s.

Twelve (33%) of the systematic reviews reported results from one type of healthcare professional, and included the following: nurses (1 review; 3%), anesthesiologists (1 review; 3%), dentists (2 reviews; 6%), general practitioners (5 reviews; 14%), community pharmacists (1 review; 3%), health workers (1 review; 3%), and mental health workers (1 review; 3%). Twenty (56%) of the reviews reported findings from multiple healthcare professional groups, and 4 reviews (11%) did not report healthcare professional group in their inclusion criteria.

The specific behaviors addressed in the systematic reviews were as follows: alcohol intake (2 reviews; 6%), diet (1 review; 3%), obesity or weight management (6 reviews; 17%), physical activity (5 reviews; 14%), physical activity and diet (1 review; 3%), smoking cessation (13 reviews; 36%), and health promotion generally (no target behavior specified: 8 reviews; 22%).

### Overall Findings

**Fig. 2:**
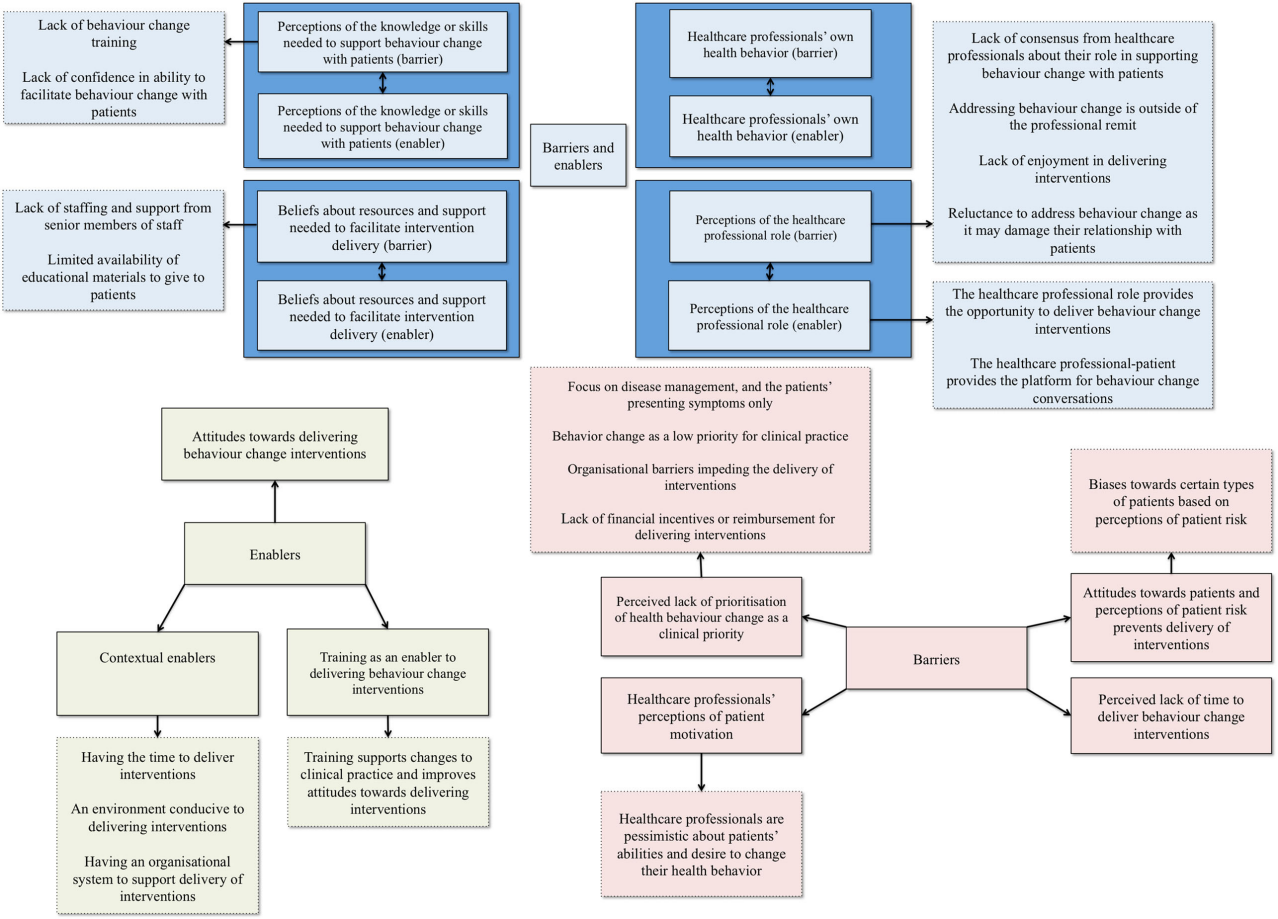
Conceptual diagram of the key findings of the present review, with relevant evidence obtained

Results are presented according to three groups of themes: (1) themes constituting both barriers and enablers to delivering behavior change interventions, (2) themes constituting unique barriers to delivering interventions, and (3) themes constituting unique enablers to delivering interventions. Within each group, sub-themes are presented in order of the amount of evidence present. Figure 2 shows a conceptual diagram of the key findings of the present review. The boxes with a bold outline represent the themes, and the boxes with a dashed outline represent the prominent sub-themes. Evidence is presented alongside the themes and sub-themes. Thirty-four of the thirty-six reviews cut across multiple themes.

### Themes Constituting Barriers and Enablers to Delivering Behavior Change Interventions

#### Perceptions of the Knowledge or Skills Needed to Support Behavior Change with Patients

Twenty systematic reviews showed healthcare professionals perceived they lacked the skills or knowledge of the available resources to help facilitate behavior change, for example, patient information sources which included signposting to other support services (Huijg et al. 2015). This was the case across diverse disciplines and specialisms, including smoking cessation and weight management support during pregnancy (Baxter et al. 2010; Heslehurst et al. 2014), smoking cessation delivered by nurses as part of cancer care (Cooley, Lundin, & Murray 2009), smoking cessation delivered by anesthesiologists (Yousefzadeh et al. 2016) and GPs (Stead et al. 2009), and for GPs to deliver diet and physical activity interventions (Bock et al. 2012). A perceived lack of skills prevented healthcare professionals from addressing physical activity in six reviews, four of which focused on studies based in primary care settings (Gentry et al. 2017).

Six of the twenty systematic reviews cited a perceived lack of behavior change training (Conlon et al. 2017). Seven of the twenty reviews reported that healthcare professionals perceived a lack of confidence in their ability to facilitate positive behavior changes in patients (Vogt et al. 2005). In addition, Johnson et al. (2011) cited a lack of awareness of recommended guidelines in relation to patient care (Johnson et al. 2011), and Cooley et al. (2009) cited a perceived lack of information by nurses to deliver smoking cessation interventions

Conversely, six systematic reviews found that healthcare professionals perceived having the right skillset to deliver behavior change interventions as an important enabler to professional practice (Baxter et al. 2010). These skills included interpersonal and counseling skills, such as being sensitive during behavior change discussions, and being able to assess patients’ motivation to change (Flemming et al. 2016), being non-judgmental towards patients (Baxter et al. 2010), and using a more persuasive, sensitive communication style, rather than “preaching” (Baxter et al. 2010).

#### Perceptions of the Healthcare Professional Role

Twelve systematic reviews highlighted the healthcare professional role as being a barrier to delivering behavior change interventions, both in relation to perceived responsibilities and the routines within each specialism (Vine et al. 2013). For example, differences in day-to-day routine practice between professionals influenced the likelihood of smoking cessation practice, such as recording smoking status, referral rates, and the general ethos in relation to behavior change practices (Baxter et al. 2010).

Restrictions due to the healthcare professional role included a lack of consensus from healthcare professionals about their role in supporting behavior change with patients (van Dillen and Hiddink 2014), perceptions that addressing behavior change was outside of the professional remit (Stead et al. 2009), a perceived lack of enjoyment in delivering behavior change interventions (three of the twelve reviews; evident among nurses (Verhaeghe et al. 2011) and GPs (Teixeira et al. 2012; Stead et al. 2009), and a reluctance to address behavior change as it may damage their relationship with patients (three of the twelve reviews; Heslehurst et al. 2014).

Across ten of the included systematic reviews, the role of the healthcare professional was seen to facilitate the delivery of behavior change interventions (Teixeira et al. 2012). Reviews in relation to pharmacists, GPs, midwives, and nurses all reported that healthcare professionals held positive views about the importance of behavior change interventions within their role.

The healthcare professionals’ role was perceived to provide the opportunity to deliver behavior change interventions in five systematic reviews (Baxter et al. 2010). For example, two reviews highlighted pregnancy as an opportune moment to intervene with smoking cessation advice (Baxter et al. 2010; Flemming et al. 2016), and Yousefzadeh et al. concluded that anesthesiologists have the opportunity to address smoking cessation with patients, even in light of the limited contact time anesthesiologists have with patients (Yousefzadeh et al. 2016).

Two of the twelve systematic reviews (one in relation to GPs and one in relation to pharmacists) emphasized the importance of the healthcare professional-patient relationship in providing the platform for professionals to talk to patients about health behavior change (Anderson et al. 2003; Dewhurst et al. 2017). Patients that are known to healthcare professionals through regular contact (Anderson et al. 2003) and increased rapport, continuity of care, and knowledge of patient health history increased the likelihood of behavior change discussion (Dewhurst et al. 2017).

#### Beliefs About Resources and Support Needed to Facilitate Intervention Delivery

Thirteen systematic reviews highlighted healthcare professionals’ perceived lack of support and resources to deliver behavior change interventions (Wandell et al. 2018). Examples included a lack of staffing and support from more senior members of staff (Guydish et al. 2007) and limited availability of educational materials to give to patients (Hebert et al. 2012). Other specific barriers included a lack of behavior-specific resources, such as available medical management options for obesity (Dewhurst et al. 2017), a lack of on-site/specialist smoking cessation services (Guydish et al. 2007; Stead et al. 2009), and a lack of physical activity support services (Heslehurst et al. 2014; Huijg et al. 2015).

Eight systematic reviews emphasized the importance placed by healthcare professionals on having access to appropriate resources and/or support from colleagues as an enabler to delivering behavior change interventions (Vine et al. 2013). Specific resources included access to appropriate physical activity interventions in primary care focusing on prevention (Huijg et al. 2015), and the perceived importance of access to smoking cessation interventions to facilitate behavior change interventions delivered by dentists as part of routine practice (Rosseel et al. 2012). Desired resources included internet-delivered support that could be provided by dental professionals about smoking cessation advice, or information about promoting a healthy weight during pregnancy for healthcare professionals working in primary care settings (Vine et al. 2013). In addition, obtaining knowledge regarding childhood obesity, for example, from medical journals, and engaging in continuing professional development were perceived to facilitate intervention delivery (Van Gerwen et al. 2009). Having a structured approach to delivering behavior interventions was seen as having a positive influence on professional practice. Examples included having access to professional guidelines (Van Gerwen et al. 2009), or an individual case management approach (Vine et al. 2013) for the prevention and management of childhood obesity, and having structured protocols for smoking cessation advice (Rosseel et al. 2012). Additionally, Lala et al. concluded that support from colleagues was perceived to be important, where professionals working individually, compared with those having the support of a team, were more likely to perceive barriers to delivering behavior change interventions (Lala et al. 2017).

#### Healthcare Professionals’ Own Health Behavior

Ten systematic reviews made reference to healthcare professionals’ own health behavior being perceived as a barrier to conveying information to patients about health behavior (Guydish et al. 2007). In two of the ten reviews, both focused on GPs, smoking status was not always linked to their smoking cessation practices (Duaso et al. 2014; Stead et al. 2009). However, eight of the ten reviews found that healthcare professionals who smoked themselves believed this could act as a barrier to delivering smoking cessation advice to their patients (Conlon et al. 2017).

Evidence for the influence of healthcare professionals’ own weight as a potential barrier to intervention delivery was less certain. Only one systematic review (of 11 quantitative studies, mostly consisting of cross-sectional surveys [*n* = 10]) found that healthcare professionals of normal weight were more likely to hold negative attitudes towards obese patients, compared with healthcare professionals who were overweight or obese (Zhu et al. 2011). However, the review did not examine the effects of negative attitudes on the delivery of interventions. Heslehurst et al.’s meta-synthesis of 25 studies (both qualitative and quantitative), in relation to the implementation of pregnancy weight management guidelines, concluded that there was no consistent pattern in whether healthcare professionals’ own weight affected communication or perceived barriers to communication (Heslehurst et al. 2014). It was also important to note that two systematic reviews, which included a meta-analysis, and a review of both qualitative and quantitative studies, found that there was no evidence for healthcare professionals’ alcohol consumption or physical activity levels acting as a barrier to the delivery of interventions.

Three systematic reviews reported that healthcare professionals’ behavior had an enabling influence on the likelihood of delivering behavior change interventions (Fie et al. 2013). Specifically, healthcare professionals reported that positive health behaviors were perceived to positively influence their professional practice in relation to delivering interventions. A review of GPs’ smoking status found that non-smoking GPs were more likely to address smoking cessation with patients, compared with GPs who smoked (Stead et al. 2009). Similar findings were obtained in two of the three reviews about healthcare professionals’ physical activity levels. Fie et al.’s review of thirteen studies found that higher physical activity levels reported by GPs and nurses were associated with higher physical activity–promoting practices (Fie et al. 2013). Hebert et al.’s review of healthcare professionals working in primary care found that physical activity advice was more likely among healthcare professionals who were active themselves (Hebert et al. 2012).

### Themes Constituting Unique Barriers to Delivering Behavior Change Interventions

#### Perceived Lack of Time to Deliver Behavior Change Interventions

Seventeen systematic reviews reported that time was perceived as a barrier to delivering behavior change interventions during consultations (Dewhurst et al. 2017). This finding was consistent across professional groups and health behaviors, including weight management (Dewhurst et al. 2017), smoking cessation (Vogt et al. 2005), physical activity (Huijg et al. 2015), improving diet (Lucas et al. 2014), and improving health behavior generally (van Dillen and Hiddink 2014). Specific reasons for this included a reported eagerness to fit with the patients’ agendas (Dewhurst et al. 2017), and in some specialisms such as anesthesiologists, only seeing patients for a limited period of time before surgery (Yousefzadeh et al. 2016).

#### Perceived Lack of Prioritization of Health Behavior Change as a Clinical Priority

Fifteen systematic reviews suggested there was a lack of prioritization, both personally and in relation to the ethos of the organization in which healthcare professionals worked, in relation to delivering behavior change interventions (van Dillen and Hiddink 2014). Five of the fifteen reviews highlighted behavior change as a low priority for clinical practice (Eakin et al. 2005). Specific reasons for this included the perception that discussing behavior change was inappropriate (Hebert et al. 2012; Vogt et al. 2005), and behavior change was not perceived as an important health risk factor, as was the case with physical activity interventions delivered in primary care (Eakin et al. 2005).

Five of the fifteen reviews highlighted a tendency for healthcare professionals to focus on disease management, and the patients’ presenting symptoms only (Verhaeghe et al. 2011). For example, smoking cessation advice is more likely provided in the presence of smoking-related symptoms (Stead, Angus, Holme, Cohen, Tait, Peña, et al., 2009), physical activity advice is more likely in the presence of cardiovascular disease (CVD)–related symptoms (Hebert et al. 2012), and weight management advice in the presence of comorbidities (Dewhurst et al. 2017). Six of the fifteen reviews identified organizational barriers as impeding the delivery of interventions (Hebert et al. 2012), such as the perception that management expectations focused on other areas of clinical practice, rather than behavior change interventions (Heslehurst et al. 2014). A lack of financial incentives or reimbursement for delivering interventions was emphasized in six of the fifteen reviews (Eakin et al. 2005).

#### Attitudes Towards Patients and Perceptions of Patient Risk Prevents Delivery of Interventions

The findings from eleven systematic reviews suggested that healthcare professionals’ attitudes towards patients and delivering behavior change interventions influenced the likelihood of intervention delivery (Eakin et al. 2005). Biases towards certain types of patients based on perceptions of patient risk were evidenced in three of the eleven reviews (Huijg et al. 2015). For example, GPs are more likely to intervene with heavy smokers than light smokers (Stead et al. 2009), and with patients whom they deemed to be at higher risk of cardiovascular disease (Bock et al. 2012), thus missing opportunities for wider scale prevention strategies with a higher number of patients. While physical activity interventions were more often delivered to patients of high socio-economic status and patients with chronic conditions, the relationship between intervention delivery and other demographics factors including income level and gender was inconclusive (Huijg et al. 2015).

Negative attitudes were present both in relation to (1) the perceived benefits of behavior change interventions, such as physical activity interventions (Huijg et al. 2015), (2) more generally towards the benefits of behavior change on patients’ health, which affected the likelihood of delivering interventions, and (3) towards patients, as was the case in patients who were obese (Heslehurst et al. 2014; Teixeira et al. 2012).

Two of the eleven reviews concluded that primary care healthcare professionals’ uncertainty about the effectiveness of physical activity interventions acted as a barrier to delivering interventions (Hebert et al. 2012; Huijg et al. 2015). Five of the eleven reviews suggested that healthcare professionals did not believe behavior change interventions would be successful in changing patients’ behavior (Dewhurst et al. 2017).

#### Healthcare Professionals’ Perceptions of Patient Motivation

Nine systematic reviews found that healthcare professionals’ perceptions of how motivated patients were to change, regardless of patient demographics and the presence or absence of chronic illness, influenced the likelihood of them addressing behavior change during the consultation (Conlon et al. 2017). Generally, healthcare professionals were pessimistic about patients’ abilities and desire to change their health behavior, which consequently affected the likelihood of delivering interventions (Conlon et al. 2017).

Examples include detrimental beliefs about obese patients, such as beliefs about patients being unmotivated, lacking self-control, and not having the ability to lose weight which resulted in inconsistencies in whether healthcare professionals delivered behavior change interventions (Teixeira et al. 2012). Similarly, a review of 25 studies (qualitative and quantitative) reporting healthcare professionals’ obesity management practices among pregnant women found that healthcare professionals believed patients lacked the willpower and motivation to lose weight (Heslehurst et al. 2014). A review of 16 qualitative studies found that physicians believed that weight management was too difficult for patients to achieve and maintain (Dewhurst et al. 2017). A review of primary care–based physical activity interventions found that in 7 of the 8 included studies, physicians believed that patients were not interested or willing to follow physical activity advice (Eakin 2005).

### Themes Constituting Unique Enablers to Delivering Behavior Change Interventions

#### Training as an Enabler to Delivering Behavior Change Interventions

Eleven systematic reviews concluded that healthcare professionals perceived appropriate training as an enabler to introducing behavior change and delivering interventions during routine consultations. This was present in reviews in relation to pharmacists, midwives, nurses, GPs, and dentists (Rosseel et al. 2012). Being able to acquire specific knowledge and skills was perceived as important across behaviors and included a perceived need to acquire weight-related communication skills (Heslehurst et al. 2014), skills to deliver brief alcohol interventions (Johnson et al. 2011), to support patients in attempts to quit smoking (Rosseel et al. 2012; Thompson et al. 2011), or specific behavior change techniques including goal setting (Vine et al. 2013).

Four of the eleven systematic reviews identified the positive influence that training had in supporting healthcare professionals to deliver interventions both in terms of improving attitudes towards delivering interventions, and also supporting changes to clinical practice (Thompson et al. 2011). This included helping pharmacists identify opportunities to support patients to make positive behavior changes, beyond customers with whom they already had regular contact (Anderson et al. 2003). Stead et al. reported an association between GPs participating in specialist smoking cessation training, and smoking cessation practices (Stead et al. 2009), and Thompson et al. (2011) concluded that healthcare professionals reported being more comfortable talking to pregnant women about smoking cessation following specialist training.

#### Contextual Enablers

Nine reviews highlighted context-specific factors that enabled delivery of behavior change interventions to patients: having the time to deliver interventions (Lala et al. 2017), working in an environment perceived to be conducive to delivering interventions (Johnson et al. 2011), and having an organizational system to support delivery of behavior change interventions (Gentry et al. 2017). Yousefzadeh et al. concluded that specialist anesthesiologists have the opportunity to deliver a brief intervention for smoking cessation, despite this only being part of a brief preoperative clinical encounter (Yousefzadeh et al. 2016). Lala et al. found that dentists who perceived themselves as having more available time were more likely to discuss smoking cessation with patients, and were more likely to signpost patients to relevant support services (Lala et al. 2017).

Two systematic reviews emphasized the importance of having an environment that enabled discussions about behavior change, such as a quiet consultation area (Anderson et al. 2003), or a specialist clinic (Johnson et al. 2011). Two reviews suggested that improving access to and awareness of available services resulted in increased likelihood of delivery of behavior change interventions (Flemming et al. 2016; Vine et al. 2013).

#### Attitudes Towards Delivering Behavior Change Interventions

Seven systematic reviews outlined the importance of positive attitudes of healthcare professionals concerning the value of behavior change interventions (Cooley et al. 2009). Van Gerwen et al. found that all GPs in the included studies thought that it was important to address childhood obesity management among both overweight and obese children as well as their parents (Van Gerwen et al. 2009). Two of the seven reviews (conducted amongst primary care professionals) concluded that positive attitudes towards physical activity enhanced physical activity promotion practices (Fie et al. 2013; Huijg et al. 2015). Similar findings were obtained for alcohol-related health promotion, where a positive association was found between attitudes towards alcohol and professional practice (Bakhshi and While 2014). Cooley et al. found that nurses’ attitudes towards delivering smoking cessation interventions were positively associated with their professional practice (Cooley et al. 2009).

### Reported Limitations of the Evidence Base

The authors of most of the included systematic reviews highlighted the limitations of the evidence base. Five reviews stated that low numbers of studies meant that drawing conclusions was difficult (Bakhshi and While 2014; Flemming et al. 2016; Lala et al. 2017; Rosseel et al. 2012; Vogt et al. 2005), and four reviews stated that the included studies had low numbers of participants (Anderson et al. 2003; Cooley et al. 2009; Stead et al. 2009; Thompson et al. 2011). Three reviews indicated that samples were often not nationally representative, making the generalization of conclusions difficult (Anderson et al. 2003; Teixeira et al. 2012; van Dillen and Hiddink 2014). One review highlighted a tendency for studies to focus on the efficacy of interventions, rather than examining practical issues in relation to implementation and generalizability of interventions (Eakin et al. 2005). The inclusion of self-report, cross-sectional data was acknowledged as a limitation in seven studies (Alonso-Perales et al. 2017; Fie et al. 2013; Hebert et al. 2012; Lala et al. 2017; Lucas et al. 2014; Stead et al. 2009; Zhu et al. 2011). In relation to self-report questionnaires, in three systematic reviews, only one study included a psychometric assessment (i.e. the reliability and validity) of the questionnaires used: 1/18 studies (Hebert et al. 2012), 1/11 studies (Teixeira et al. 2012), and 1/11 (Zhu et al. 2011) studies respectively. Two systematic reviews highlighted that using single methods of data collection was problematic, and that using multiple methods (i.e., qualitative and quantitative methods) would allow for triangulation, consequently adding strength and depth to study findings (Baxter et al. 2010; Teixeira et al. 2012). Two systematic reviews suggested study designs often neglected a qualitative component (Gentry et al. 2017; Teixeira et al. 2012).

## Discussion

This is the first systematic review of systematic reviews to synthesize the cross-disciplinary barriers and enablers to delivering behavior change advice across all healthcare professional disciplines. There were four unique barriers to delivering behavior change advice across diverse healthcare professional disciplines: (1) attitudes towards patients, (2) perceptions of patient motivation, (3) perceived lack of time, and (4) perceived lack of prioritization. There were three unique enablers: (1) attitudes towards delivering advice, (2) the importance of training, and (3) contextual enablers. Four factors constituted both barriers and enablers: (1) healthcare professionals’ own health behavior, (2) the healthcare professional role, (3) knowledge and skills, and (4) resources and support. Based on our findings, there are three key areas for future interventions. First, to address healthcare professionals’ perceptions about the healthcare professional role in delivering interventions and patient need for behavior change interventions. Second, to support healthcare professionals to identify opportunities to deliver interventions during routine practice. Third, to deliver training targeting the cross-disciplinary barriers and enablers identified in our review. The thirty-six systematic reviews included in the present review provide a comprehensive overview of the factors involved in implementing this area of clinical practice.

Our review extends the findings from previous systematic reviews by, for the first time, demonstrating that the barriers and enablers previously reported in discipline-specific reviews, including a perceived lack of time (Heslehurst, Newham, et al. 2014), and a lack of knowledge and skills (Yousefzadeh et al. 2016) in relation to delivering behavior change interventions, are shared across diverse professional groups. This consequently provides the opportunities to: (1) develop interventions and inform training that target specific areas of clinical practice known to impede or improve the delivery of interventions, and (2) facilitate the implementation of public health policies that are used internationally to encourage healthcare professionals to incorporate behavior change interventions into routine consultations (Public Health England 2016; Whitlock et al. 2002).

Our systematic review of systematic reviews identified barriers in relation to perceptions of patient motivation in relation to behavior change (Guydish et al. 2007), and healthcare professionals’ attitudes towards patients (Teixeira et al. 2012). There is a commonly held notion, highlighted by our findings, that healthcare professionals believe that patients (a) do not want or need information about behavior change, and patients would find this information inappropriate (Hebert et al. 2012; Vogt et al. 2005); and (b) lack the motivation or desire, and were not willing to make sustained changes (Dewhurst et al. 2017). Consequently, healthcare professionals make conscious decisions about delivering behavior change advice based on perceptions of patient risk and patient motivation to take preventative action (Bonner et al. 2015), rather than based on patient need.

The current evidence base demonstrates an urgent need to address behavior change during routine medical consultations. The recent health survey for England showed that 32% of patients reported having two out of five behavioral risk factors (in relation to smoking, excess alcohol, diet, physical inactivity, and weight); 19% had three out of five (NHS Digital 2018). Despite this, a recent survey found that healthcare professionals reported delivering behavior change interventions to just 50% of patients who they perceived would benefit from an intervention (Keyworth et al. 2019). Thus, the evidence suggests a missed opportunity to deliver behavior change to patients who would benefit, which consequently meets the remit of public health policies, which are designed to encourage healthcare professionals to deliver opportunistic behavior change advice to patients (reference blinded). Our review suggests that facilitating healthcare professional practice with known enablers includes having access to the relevant resources and support to facilitate intervention delivery (Rosseel et al. 2012; Van Gerwen et al. 2009), and an environment that is conducive to providing behavior change support (Johnson et al. 2011).

### Strengths and Limitations of This Review

We were able to (1) synthesize all systematic reviews examining the barriers and enablers to healthcare professionals’ delivery of behavior change advice, and (2) identify the barriers and enablers shared across diverse professional groups. This review is timely due to the wealth of published literature in this area (our review of 36 reviews included 958 unique studies, with a mean overlap of 16.92%; thirteen reviews [36.1%] had no overlap). We have provided a much-needed synthesis of this work with the key aim of identifying a set of barriers and enablers shared across disciplines, which are likely to enable the implementation of training interventions to be delivered at scale.

A potential limitation of the present systematic review is the heterogeneity of the included reviews, which was highlighted by review authors (Conlon et al. 2017; Crisford et al. 2018; Kelly et al. 2017; Knudsen 2017), and may suggest a need for more consistent reporting. A note of caution must therefore be added when interpreting the results of this review, given the mean AMSTAR score of included reviews was 3.8/7. Specific areas for improvement based on our ratings include ensuring authors publish their study protocols or register their review to ensure an “a priori” design, and ensuring authors make clear their search strategy includes a gray literature search. Two systematic reviews among the lowest scoring in our review (Knudsen 2017; Verhaeghe et al. 2011) failed to use a comprehensive search strategy (at least two electronic sources). Consequently, relevant studies may have been missed, which may prevent a comprehensive assessment of the evidence base. Conversely, the highest scoring reviews (Crisford et al. 2018; Duaso et al. 2014) used a comprehensive search strategy, assessed the scientific quality of included studies, as well as making explicit statements about study quality when formulating subsequent conclusions, and making recommendations for future research and practice.

The narrative synthesis conducted on the data allowed for (1) a rich insight into the barriers and enablers to delivering interventions, and (2) to summarize a complex body of literature into a series of recommendations that can facilitate implementation of behavior change into healthcare professional practice.

## Conclusions

The major factors extracted and synthesized from this review suggest that two key areas can be targeted for future interventions. First, to address healthcare professionals’ perceptions about patient need for behavior change interventions, and to facilitate healthcare professionals to identify opportunities to deliver interventions during routine practice. Second, to provide training to address the barriers identified in this review across diverse professional groups. By widening the scope of the consultation, patients can be considered in a broader way, focusing on prevention *and* the management of health conditions. Healthcare professionals are well placed to support behavior change with patients, and our review suggests there are opportunities to facilitate this important area of medical practice.

### Funding Information

This study was funded by a research grant obtained from Tesco Plc (grant number R119456) and supported by the NIHR Manchester Biomedical Research Centre and NIHR Greater Manchester Patient Safety Translational Research Centre. Tesco and NIHR had no role in the design of this study and did not have any role during its execution, analyses, interpretation, and storage of the data or decision to submit results.

## Electronic Supplementary Material

ESM 1 (DOCX 146 kb)

ESM 2 (DOCX 105 kb)

ESM 3 (DOCX 106 kb)
