# Supplementary material for: Delivering Opportunistic Behavior Change Interventions: a Systematic Review of Systematic Reviews
Source: Prev Sci. 2020 Feb 17;21(3):319–31. doi: 10.1007/s11121-020-01087-6 (PMC7056685; doi:10.1007/s11121-020-01087-6)
Supplement: Supplementary file 1 — (DOCX 146 kb) [file 11121_2020_1087_MOESM1_ESM.docx]

**Supplementary File A: Search strategies**

**PubMed:**

(((("health personnel"[MeSH Terms] AND "health promotion"[MeSH Terms]) OR "health communication"[MeSH Terms]) OR "health education"[MeSH Terms]) AND weight[Title/Abstract] OR "physical activity"[Title/Abstract] OR exercise[Title/Abstract] OR "smoking"[Title/Abstract] OR “smoking cessation”[MeSH Terms] OR “weight loss”[MeSH Terms] OR exercise[MeSH Terms] OR diet[MeSH Terms] OR "Alcohol Drinking"[Mesh] AND (barriers[Title/Abstract] OR facilitators[Title/Abstract] OR beliefs[Title/Abstract] OR attitudes[Title/Abstract] OR opinions[Title/Abstract] OR perceptions[Title/Abstract])) AND (meta-analysis[Title/Abstract] OR synthesis[Title/Abstract] OR review[Title/Abstract]) AND Review[ptyp] AND Review[ptyp] AND ("humans"[MeSH Terms] AND English[lang]) NOT protocol[Title/Abstract] NOT "drug use"[Title/Abstract] NOT "medication adherence"[Title/Abstract]

**CINAHL:**

| [**Search ID#**](javascript:__doPostBack('ctl00$ctl00$MainContentArea$MainContentArea$historyControl$ReorderHistoryLink','')) | **Search Terms** | **Search Options** | **Actions** |  |
| --- | --- | --- | --- | --- |
|  | S26 | (S15 OR S16 OR S17 OR S18) AND (S1 AND S22 AND S23 AND S24 AND S25) | Search modes - Boolean/Phrase | [Rerun](javascript:__doPostBack('ctl00$ctl00$MainContentArea$MainContentArea$historyControl$HistoryRepeater$ctl00$linkResults',''))  [View Details](javascript:showShDetails(%22ctl00_ctl00_MainContentArea_MainContentArea_historyControl_ctrlPopup%22,%20%22S26%22);)  [Edit](http://web.b.ebscohost.com/Legacy/Views/UserControls/Ehost/) |
|  | S25 | S15 OR S16 OR S17 OR S18 | Search modes - Boolean/Phrase | [Rerun](javascript:__doPostBack('ctl00$ctl00$MainContentArea$MainContentArea$historyControl$HistoryRepeater$ctl01$linkResults',''))  [View Details](javascript:showShDetails(%22ctl00_ctl00_MainContentArea_MainContentArea_historyControl_ctrlPopup%22,%20%22S25%22);)  [Edit](http://web.b.ebscohost.com/Legacy/Views/UserControls/Ehost/) |
|  | S24 | S9 OR S10 OR S11 OR S12 OR S13 OR S14 | Search modes - Boolean/Phrase | [Rerun](javascript:__doPostBack('ctl00$ctl00$MainContentArea$MainContentArea$historyControl$HistoryRepeater$ctl02$linkResults',''))  [View Details](javascript:showShDetails(%22ctl00_ctl00_MainContentArea_MainContentArea_historyControl_ctrlPopup%22,%20%22S24%22);)  [Edit](http://web.b.ebscohost.com/Legacy/Views/UserControls/Ehost/) |
|  | S23 | S5 OR S6 OR S7 OR S8 OR S20 OR S21 | Search modes - Boolean/Phrase | [Rerun](javascript:__doPostBack('ctl00$ctl00$MainContentArea$MainContentArea$historyControl$HistoryRepeater$ctl03$linkResults',''))  [View Details](javascript:showShDetails(%22ctl00_ctl00_MainContentArea_MainContentArea_historyControl_ctrlPopup%22,%20%22S23%22);)  [Edit](http://web.b.ebscohost.com/Legacy/Views/UserControls/Ehost/) |
|  | S22 | S2 OR S3 OR S4 | Search modes - Boolean/Phrase | [Rerun](javascript:__doPostBack('ctl00$ctl00$MainContentArea$MainContentArea$historyControl$HistoryRepeater$ctl04$linkResults',''))  [View Details](javascript:showShDetails(%22ctl00_ctl00_MainContentArea_MainContentArea_historyControl_ctrlPopup%22,%20%22S22%22);)  [Edit](http://web.b.ebscohost.com/Legacy/Views/UserControls/Ehost/) |
|  | S21 | (MH "Alcohol Drinking+") OR "alcohol" | Search modes - Boolean/Phrase | [Rerun](javascript:__doPostBack('ctl00$ctl00$MainContentArea$MainContentArea$historyControl$HistoryRepeater$ctl05$linkResults',''))  [View Details](javascript:showShDetails(%22ctl00_ctl00_MainContentArea_MainContentArea_historyControl_ctrlPopup%22,%20%22S21%22);)  [Edit](http://web.b.ebscohost.com/Legacy/Views/UserControls/Ehost/) |
|  | S20 | MH diet | Search modes - Boolean/Phrase | [Rerun](javascript:__doPostBack('ctl00$ctl00$MainContentArea$MainContentArea$historyControl$HistoryRepeater$ctl06$linkResults',''))  [View Details](javascript:showShDetails(%22ctl00_ctl00_MainContentArea_MainContentArea_historyControl_ctrlPopup%22,%20%22S20%22);)  [Edit](http://web.b.ebscohost.com/Legacy/Views/UserControls/Ehost/) |
|  | S19 | MH exercise or physical activity | Search modes - Boolean/Phrase | [Rerun](javascript:__doPostBack('ctl00$ctl00$MainContentArea$MainContentArea$historyControl$HistoryRepeater$ctl07$linkResults',''))  [View Details](javascript:showShDetails(%22ctl00_ctl00_MainContentArea_MainContentArea_historyControl_ctrlPopup%22,%20%22S19%22);)  [Edit](http://web.b.ebscohost.com/Legacy/Views/UserControls/Ehost/) |
|  | S18 | MH systematic review | Search modes - Boolean/Phrase | [Rerun](javascript:__doPostBack('ctl00$ctl00$MainContentArea$MainContentArea$historyControl$HistoryRepeater$ctl08$linkResults',''))  [View Details](javascript:showShDetails(%22ctl00_ctl00_MainContentArea_MainContentArea_historyControl_ctrlPopup%22,%20%22S18%22);)  [Edit](http://web.b.ebscohost.com/Legacy/Views/UserControls/Ehost/) |
|  | S17 | "review" | Search modes - Boolean/Phrase | [Rerun](javascript:__doPostBack('ctl00$ctl00$MainContentArea$MainContentArea$historyControl$HistoryRepeater$ctl09$linkResults',''))  [View Details](javascript:showShDetails(%22ctl00_ctl00_MainContentArea_MainContentArea_historyControl_ctrlPopup%22,%20%22S17%22);)  [Edit](http://web.b.ebscohost.com/Legacy/Views/UserControls/Ehost/) |
|  | S16 | (MH "Meta Synthesis") OR "meta synthesis" | Search modes - Boolean/Phrase | [Rerun](javascript:__doPostBack('ctl00$ctl00$MainContentArea$MainContentArea$historyControl$HistoryRepeater$ctl10$linkResults',''))  [View Details](javascript:showShDetails(%22ctl00_ctl00_MainContentArea_MainContentArea_historyControl_ctrlPopup%22,%20%22S16%22);)  [Edit](http://web.b.ebscohost.com/Legacy/Views/UserControls/Ehost/) |
|  | S15 | (MH "Meta Analysis") OR "meta analysis" | Search modes - Boolean/Phrase | [Rerun](javascript:__doPostBack('ctl00$ctl00$MainContentArea$MainContentArea$historyControl$HistoryRepeater$ctl11$linkResults',''))  [View Details](javascript:showShDetails(%22ctl00_ctl00_MainContentArea_MainContentArea_historyControl_ctrlPopup%22,%20%22S15%22);)  [Edit](http://web.b.ebscohost.com/Legacy/Views/UserControls/Ehost/) |
|  | S14 | "perceptions" | Search modes - Boolean/Phrase | [Rerun](javascript:__doPostBack('ctl00$ctl00$MainContentArea$MainContentArea$historyControl$HistoryRepeater$ctl12$linkResults',''))  [View Details](javascript:showShDetails(%22ctl00_ctl00_MainContentArea_MainContentArea_historyControl_ctrlPopup%22,%20%22S14%22);)  [Edit](http://web.b.ebscohost.com/Legacy/Views/UserControls/Ehost/) |
|  | S13 | "opinions" | Search modes - Boolean/Phrase | [Rerun](javascript:__doPostBack('ctl00$ctl00$MainContentArea$MainContentArea$historyControl$HistoryRepeater$ctl13$linkResults',''))  [View Details](javascript:showShDetails(%22ctl00_ctl00_MainContentArea_MainContentArea_historyControl_ctrlPopup%22,%20%22S13%22);)  [Edit](http://web.b.ebscohost.com/Legacy/Views/UserControls/Ehost/) |
|  | S12 | "attitudes" OR (MH "Attitude of Health Personnel+") | Search modes - Boolean/Phrase | [Rerun](javascript:__doPostBack('ctl00$ctl00$MainContentArea$MainContentArea$historyControl$HistoryRepeater$ctl14$linkResults',''))  [View Details](javascript:showShDetails(%22ctl00_ctl00_MainContentArea_MainContentArea_historyControl_ctrlPopup%22,%20%22S12%22);)  [Edit](http://web.b.ebscohost.com/Legacy/Views/UserControls/Ehost/) |
|  | S11 | "beliefs" | Search modes - Boolean/Phrase | [Rerun](javascript:__doPostBack('ctl00$ctl00$MainContentArea$MainContentArea$historyControl$HistoryRepeater$ctl15$linkResults',''))  [View Details](javascript:showShDetails(%22ctl00_ctl00_MainContentArea_MainContentArea_historyControl_ctrlPopup%22,%20%22S11%22);)  [Edit](http://web.b.ebscohost.com/Legacy/Views/UserControls/Ehost/) |
|  | S10 | "facilitators" | Search modes - Boolean/Phrase | [Rerun](javascript:__doPostBack('ctl00$ctl00$MainContentArea$MainContentArea$historyControl$HistoryRepeater$ctl16$linkResults',''))  [View Details](javascript:showShDetails(%22ctl00_ctl00_MainContentArea_MainContentArea_historyControl_ctrlPopup%22,%20%22S10%22);)  [Edit](http://web.b.ebscohost.com/Legacy/Views/UserControls/Ehost/) |
|  | S9 | "barriers" | Search modes - Boolean/Phrase | [Rerun](javascript:__doPostBack('ctl00$ctl00$MainContentArea$MainContentArea$historyControl$HistoryRepeater$ctl17$linkResults',''))  [View Details](javascript:showShDetails(%22ctl00_ctl00_MainContentArea_MainContentArea_historyControl_ctrlPopup%22,%20%22S9%22);)  [Edit](http://web.b.ebscohost.com/Legacy/Views/UserControls/Ehost/) |
|  | S8 | (MH "Smoking+") OR "smoking" OR (MH "Smoking Cessation") | Search modes - Boolean/Phrase | [Rerun](javascript:__doPostBack('ctl00$ctl00$MainContentArea$MainContentArea$historyControl$HistoryRepeater$ctl18$linkResults',''))  [View Details](javascript:showShDetails(%22ctl00_ctl00_MainContentArea_MainContentArea_historyControl_ctrlPopup%22,%20%22S8%22);)  [Edit](http://web.b.ebscohost.com/Legacy/Views/UserControls/Ehost/) |
|  | S7 | "exercise" | Search modes - Boolean/Phrase | [Rerun](javascript:__doPostBack('ctl00$ctl00$MainContentArea$MainContentArea$historyControl$HistoryRepeater$ctl19$linkResults',''))  [View Details](javascript:showShDetails(%22ctl00_ctl00_MainContentArea_MainContentArea_historyControl_ctrlPopup%22,%20%22S7%22);)  [Edit](http://web.b.ebscohost.com/Legacy/Views/UserControls/Ehost/) |
|  | S6 | "physical activity" | Search modes - Boolean/Phrase | [Rerun](javascript:__doPostBack('ctl00$ctl00$MainContentArea$MainContentArea$historyControl$HistoryRepeater$ctl20$linkResults',''))  [View Details](javascript:showShDetails(%22ctl00_ctl00_MainContentArea_MainContentArea_historyControl_ctrlPopup%22,%20%22S6%22);)  [Edit](http://web.b.ebscohost.com/Legacy/Views/UserControls/Ehost/) |
|  | S5 | "weight" OR (MH "Weight Control") | Search modes - Boolean/Phrase | [Rerun](javascript:__doPostBack('ctl00$ctl00$MainContentArea$MainContentArea$historyControl$HistoryRepeater$ctl21$linkResults',''))  [View Details](javascript:showShDetails(%22ctl00_ctl00_MainContentArea_MainContentArea_historyControl_ctrlPopup%22,%20%22S5%22);)  [Edit](http://web.b.ebscohost.com/Legacy/Views/UserControls/Ehost/) |
|  | S4 | (MH "Health Education+") OR "health education" | Search modes - Boolean/Phrase | [Rerun](javascript:__doPostBack('ctl00$ctl00$MainContentArea$MainContentArea$historyControl$HistoryRepeater$ctl22$linkResults',''))  [View Details](javascript:showShDetails(%22ctl00_ctl00_MainContentArea_MainContentArea_historyControl_ctrlPopup%22,%20%22S4%22);)  [Edit](http://web.b.ebscohost.com/Legacy/Views/UserControls/Ehost/) |
|  | S3 | "health communication" | Search modes - Boolean/Phrase | [Rerun](javascript:__doPostBack('ctl00$ctl00$MainContentArea$MainContentArea$historyControl$HistoryRepeater$ctl23$linkResults',''))  [View Details](javascript:showShDetails(%22ctl00_ctl00_MainContentArea_MainContentArea_historyControl_ctrlPopup%22,%20%22S3%22);)  [Edit](http://web.b.ebscohost.com/Legacy/Views/UserControls/Ehost/) |
|  | S2 | (MH "Health Promotion+") OR "health promotion" OR (MH "Health Services+") | Search modes - Boolean/Phrase | [Rerun](javascript:__doPostBack('ctl00$ctl00$MainContentArea$MainContentArea$historyControl$HistoryRepeater$ctl24$linkResults',''))  [View Details](javascript:showShDetails(%22ctl00_ctl00_MainContentArea_MainContentArea_historyControl_ctrlPopup%22,%20%22S2%22);)  [Edit](http://web.b.ebscohost.com/Legacy/Views/UserControls/Ehost/) |
|  | S1 | (MH "Health Personnel+") OR "health personnel" | Search modes - Boolean/Phrase | [Rerun](javascript:__doPostBack('ctl00$ctl00$MainContentArea$MainContentArea$historyControl$HistoryRepeater$ctl25$linkResults',''))  [View Details](javascript:showShDetails(%22ctl00_ctl00_MainContentArea_MainContentArea_historyControl_ctrlPopup%22,%20%22S1%22);)  [Edit](http://web.b.ebscohost.com/Legacy/Views/UserControls/Ehost/) |

**Embase:**

| [**# ▲**](https://ovidsp.uk.ovid.com/sp-3.25.0a/ovidweb.cgi?&S=PCBFPDLEDMHFGONHFNGKLFBGDKHLAA00&Sort+Sets=descending) | **Searches** | **Results** | **Type** | **Actions** | **Annotations** |  |
| --- | --- | --- | --- | --- | --- | --- |
|  | | | | | | |
|  | 1 | exp health care personnel/ | 1197377 | Advanced | [Display Results](https://ovidsp.uk.ovid.com/sp-3.25.0a/ovidweb.cgi?&S=PCBFPDLEDMHFGONHFNGKLFBGDKHLAA00&SELECT=S.sh%7c&R=1&Process+Action=display)  [More](https://ovidsp.uk.ovid.com/sp-3.25.0a/ovidweb.cgi) |  |
|  | 2 | exp health promotion/ | 82656 | Advanced | [Display Results](https://ovidsp.uk.ovid.com/sp-3.25.0a/ovidweb.cgi?&S=PCBFPDLEDMHFGONHFNGKLFBGDKHLAA00&SELECT=S.sh%7c&R=2&Process+Action=display)  [More](https://ovidsp.uk.ovid.com/sp-3.25.0a/ovidweb.cgi) |  |
|  | 3 | exp health education/ | 272467 | Advanced | [Display Results](https://ovidsp.uk.ovid.com/sp-3.25.0a/ovidweb.cgi?&S=PCBFPDLEDMHFGONHFNGKLFBGDKHLAA00&SELECT=S.sh%7c&R=3&Process+Action=display)  [More](https://ovidsp.uk.ovid.com/sp-3.25.0a/ovidweb.cgi) |  |
|  | 4 | weight.mp. | 1184127 | Advanced | [Display Results](https://ovidsp.uk.ovid.com/sp-3.25.0a/ovidweb.cgi?&S=PCBFPDLEDMHFGONHFNGKLFBGDKHLAA00&SELECT=S.sh%7c&R=4&Process+Action=display)  [More](https://ovidsp.uk.ovid.com/sp-3.25.0a/ovidweb.cgi) |  |
|  | 5 | "physical activity".mp. or exp physical activity/ | 345016 | Advanced | [Display Results](https://ovidsp.uk.ovid.com/sp-3.25.0a/ovidweb.cgi?&S=PCBFPDLEDMHFGONHFNGKLFBGDKHLAA00&SELECT=S.sh%7c&R=5&Process+Action=display)  [More](https://ovidsp.uk.ovid.com/sp-3.25.0a/ovidweb.cgi) |  |
|  | 6 | exp exercise/ or exercise.mp. | 398930 | Advanced | [Display Results](https://ovidsp.uk.ovid.com/sp-3.25.0a/ovidweb.cgi?&S=PCBFPDLEDMHFGONHFNGKLFBGDKHLAA00&SELECT=S.sh%7c&R=6&Process+Action=display)  [More](https://ovidsp.uk.ovid.com/sp-3.25.0a/ovidweb.cgi) |  |
|  | 7 | exp smoking/ or exp smoking cessation/ or smoking.mp. | 376905 | Advanced | [Display Results](https://ovidsp.uk.ovid.com/sp-3.25.0a/ovidweb.cgi?&S=PCBFPDLEDMHFGONHFNGKLFBGDKHLAA00&SELECT=S.sh%7c&R=7&Process+Action=display)  [More](https://ovidsp.uk.ovid.com/sp-3.25.0a/ovidweb.cgi) |  |
|  | 8 | barriers.mp. | 111486 | Advanced | [Display Results](https://ovidsp.uk.ovid.com/sp-3.25.0a/ovidweb.cgi?&S=PCBFPDLEDMHFGONHFNGKLFBGDKHLAA00&SELECT=S.sh%7c&R=8&Process+Action=display)  [More](https://ovidsp.uk.ovid.com/sp-3.25.0a/ovidweb.cgi) |  |
|  | 9 | facilitators.mp. | 10872 | Advanced | [Display Results](https://ovidsp.uk.ovid.com/sp-3.25.0a/ovidweb.cgi?&S=PCBFPDLEDMHFGONHFNGKLFBGDKHLAA00&SELECT=S.sh%7c&R=9&Process+Action=display)  [More](https://ovidsp.uk.ovid.com/sp-3.25.0a/ovidweb.cgi) |  |
|  | 10 | beliefs.mp. | 52115 | Advanced | [Display Results](https://ovidsp.uk.ovid.com/sp-3.25.0a/ovidweb.cgi?&S=PCBFPDLEDMHFGONHFNGKLFBGDKHLAA00&SELECT=S.sh%7c&R=10&Process+Action=display)  [More](https://ovidsp.uk.ovid.com/sp-3.25.0a/ovidweb.cgi) |  |
|  | 11 | exp health personnel attitude/ | 156729 | Advanced | [Display Results](https://ovidsp.uk.ovid.com/sp-3.25.0a/ovidweb.cgi?&S=PCBFPDLEDMHFGONHFNGKLFBGDKHLAA00&SELECT=S.sh%7c&R=11&Process+Action=display)  [More](https://ovidsp.uk.ovid.com/sp-3.25.0a/ovidweb.cgi) |  |
|  | 12 | opinions.mp. | 33581 | Advanced | [Display Results](https://ovidsp.uk.ovid.com/sp-3.25.0a/ovidweb.cgi?&S=PCBFPDLEDMHFGONHFNGKLFBGDKHLAA00&SELECT=S.sh%7c&R=12&Process+Action=display)  [More](https://ovidsp.uk.ovid.com/sp-3.25.0a/ovidweb.cgi) |  |
|  | 13 | perceptions.mp. | 89194 | Advanced | [Display Results](https://ovidsp.uk.ovid.com/sp-3.25.0a/ovidweb.cgi?&S=PCBFPDLEDMHFGONHFNGKLFBGDKHLAA00&SELECT=S.sh%7c&R=13&Process+Action=display)  [More](https://ovidsp.uk.ovid.com/sp-3.25.0a/ovidweb.cgi) |  |
|  | 14 | meta analysis/ | 124923 | Advanced | [Display Results](https://ovidsp.uk.ovid.com/sp-3.25.0a/ovidweb.cgi?&S=PCBFPDLEDMHFGONHFNGKLFBGDKHLAA00&SELECT=S.sh%7c&R=14&Process+Action=display)  [More](https://ovidsp.uk.ovid.com/sp-3.25.0a/ovidweb.cgi) |  |
|  | 15 | synthesis.mp. or synthesis/ | 1143643 | Advanced | [Display Results](https://ovidsp.uk.ovid.com/sp-3.25.0a/ovidweb.cgi?&S=PCBFPDLEDMHFGONHFNGKLFBGDKHLAA00&SELECT=S.sh%7c&R=15&Process+Action=display)  [More](https://ovidsp.uk.ovid.com/sp-3.25.0a/ovidweb.cgi) |  |
|  | 16 | "review"/ | 2155544 | Advanced | [Display Results](https://ovidsp.uk.ovid.com/sp-3.25.0a/ovidweb.cgi?&S=PCBFPDLEDMHFGONHFNGKLFBGDKHLAA00&SELECT=S.sh%7c&R=16&Process+Action=display)  [More](https://ovidsp.uk.ovid.com/sp-3.25.0a/ovidweb.cgi) |  |
|  | 17 | exp diet/ | 256802 | Advanced | [Display Results](https://ovidsp.uk.ovid.com/sp-3.25.0a/ovidweb.cgi?&S=PCBFPDLEDMHFGONHFNGKLFBGDKHLAA00&SELECT=S.sh%7c&R=17&Process+Action=display)  [More](https://ovidsp.uk.ovid.com/sp-3.25.0a/ovidweb.cgi) |  |
|  | 18 | alcohol intake.mp. or exp alcohol consumption/ | 104571 | Advanced | [Display Results](https://ovidsp.uk.ovid.com/sp-3.25.0a/ovidweb.cgi?&S=PCBFPDLEDMHFGONHFNGKLFBGDKHLAA00&SELECT=S.sh%7c&R=18&Process+Action=display)  [More](https://ovidsp.uk.ovid.com/sp-3.25.0a/ovidweb.cgi) |  |
|  | 19 | 2 or 3 | 272467 | Advanced | [Display Results](https://ovidsp.uk.ovid.com/sp-3.25.0a/ovidweb.cgi?&S=PCBFPDLEDMHFGONHFNGKLFBGDKHLAA00&SELECT=S.sh%7c&R=19&Process+Action=display)  [More](https://ovidsp.uk.ovid.com/sp-3.25.0a/ovidweb.cgi) |  |
|  | 20 | 4 or 5 or 6 or 7 or 17 or 18 | 2273968 | Advanced | [Display Results](https://ovidsp.uk.ovid.com/sp-3.25.0a/ovidweb.cgi?&S=PCBFPDLEDMHFGONHFNGKLFBGDKHLAA00&SELECT=S.sh%7c&R=20&Process+Action=display)  [More](https://ovidsp.uk.ovid.com/sp-3.25.0a/ovidweb.cgi) |  |
|  | 21 | 8 or 9 or 10 or 11 or 12 or 13 | 404348 | Advanced | [Display Results](https://ovidsp.uk.ovid.com/sp-3.25.0a/ovidweb.cgi?&S=PCBFPDLEDMHFGONHFNGKLFBGDKHLAA00&SELECT=S.sh%7c&R=21&Process+Action=display)  [More](https://ovidsp.uk.ovid.com/sp-3.25.0a/ovidweb.cgi) |  |
|  | 22 | 14 or 15 or 16 | 3290781 | Advanced | [Display Results](https://ovidsp.uk.ovid.com/sp-3.25.0a/ovidweb.cgi?&S=PCBFPDLEDMHFGONHFNGKLFBGDKHLAA00&SELECT=S.sh%7c&R=22&Process+Action=display)  [More](https://ovidsp.uk.ovid.com/sp-3.25.0a/ovidweb.cgi) |  |
|  | 23 | 1 and 19 and 20 and 21 and 22 | 202 | Advanced | [Display Results](https://ovidsp.uk.ovid.com/sp-3.25.0a/ovidweb.cgi?&S=PCBFPDLEDMHFGONHFNGKLFBGDKHLAA00&SELECT=S.sh%7c&R=23&Process+Action=display)  [More](https://ovidsp.uk.ovid.com/sp-3.25.0a/ovidweb.cgi) |  |
|  | 24 | limit 23 to (human and english language and "review") | 179 | Advanced | [Display Results](https://ovidsp.uk.ovid.com/sp-3.25.0a/ovidweb.cgi?&S=PCBFPDLEDMHFGONHFNGKLFBGDKHLAA00&SELECT=S.sh%7c&R=24&Process+Action=display)  [More](https://ovidsp.uk.ovid.com/sp-3.25.0a/ovidweb.cgi) |  |

**Web of Science:**

You searched for: **TOPIC:** (((“Health professional” OR “Health practitioner” OR “Health personnel” OR Anaesthetist OR Audiologist OR Chiropractor OR Dentist OR Dietician OR Dietitian OR “General practitioner” OR Doctor OR Gynaecologist OR “Health visitor” OR Midwife OR Nurse OR Obstetrician OR Ophthalmologist OR Optician OR Osteopath OR Paediatrician OR Pathologist OR Pharmacist OR Psychiatrist OR Psychologist OR Radiologist OR “Social Worker” OR “Speech therapist” OR “Language therapist” OR Surgeon OR Physician OR “Primary care” OR “Secondary care”))) *AND* **TOPIC:** ((( "Health Information" OR "Health Advice" OR "Health Intervention" OR "Service provision" OR "Behaviour change" OR "lifestyle intervention" OR Counselling OR "Health Communication" OR "Health Promotion" OR "Health education" ))) *AND* **TOPIC:** ((barriers OR facilitators OR beliefs OR attitudes OR opinions OR Perceptions)) *AND* **TOPIC:** ((Diet OR Nutrition OR Fruit OR Vegetables OR Sugar OR Fat OR Fibre OR Salt OR “Physical activity” OR Exercise OR Inactivity OR “Weight loss” OR BMI OR “Body mass index” OR “Sedentary behaviour” OR smoking OR “smoking cessation” OR alcohol OR “alcohol intake”))

**Refined by:** **DOCUMENT TYPES:** ( REVIEW )

**Timespan:** All years. **Indexes:** SCI-EXPANDED, SSCI, A&HCI, CPCI-S, CPCI-SSH, BKCI-S, BKCI-SSH, ESCI, CCR-EXPANDED, IC.

**Scopus (filtered by review)**

KEY ( "Health professional" OR "Health practitioner" OR "Health personnel" OR anaesthetist OR audiologist OR chiropractor OR dentist OR dietician OR dietitian OR "General practitioner" OR doctor OR gynaecologist OR "Health visitor" OR midwife OR nurse OR obstetrician OR ophthalmologist OR optician OR osteopath OR paediatrician OR pathologist OR pharmacist OR psychiatrist OR psychologist OR radiologist OR "Social Worker" OR "Speech therapist" OR "Language therapist" OR surgeon OR physician OR "Primary care" OR "Secondary care" ) AND KEY ( "Health Information" OR "Health Advice" OR "Health Intervention" OR "Service provision" OR "Behaviour change" OR "lifestyle intervention" OR counselling OR "Health Communication" OR "Health Promotion" OR "Health education" ) AND KEY ( barriers OR facilitators OR beliefs OR attitudes OR opinions OR perceptions ) AND KEY ( diet OR nutrition OR fruit OR vegetables OR sugar OR fat OR fibre OR salt OR "Physical activity" OR exercise OR inactivity OR "Weight loss" OR bmi OR "Body mass index" OR "Sedentary behaviour" OR smoking OR "smoking cessation" OR alcohol OR "alcohol intake" ) AND ( LIMIT-TO ( DOCTYPE , "re" ) ) AND ( LIMIT-TO ( LANGUAGE , "English" ) )

**PsycINFO:**

| [**# ▲**](http://ovidsp.uk.ovid.com/sp-3.25.0a/ovidweb.cgi?&S=FMDJPDABLJHFJNEKFNGKCAJHEDGOAA00&Sort+Sets=descending) | **Searches** | **Results** | **Type** | **Actions** | **Annotations** |  |
| --- | --- | --- | --- | --- | --- | --- |
|  | | | | | | |
|  | 1 | exp Health Personnel/ | 120741 | Advanced | [Display Results](http://ovidsp.uk.ovid.com/sp-3.25.0a/ovidweb.cgi?&S=FMDJPDABLJHFJNEKFNGKCAJHEDGOAA00&SELECT=S.sh%7c&R=1&Process+Action=display)  [More](http://ovidsp.uk.ovid.com/sp-3.25.0a/ovidweb.cgi) |  |
|  | 2 | exp Health Promotion/ | 20909 | Advanced | [Display Results](http://ovidsp.uk.ovid.com/sp-3.25.0a/ovidweb.cgi?&S=FMDJPDABLJHFJNEKFNGKCAJHEDGOAA00&SELECT=S.sh%7c&R=2&Process+Action=display)  [More](http://ovidsp.uk.ovid.com/sp-3.25.0a/ovidweb.cgi) |  |
|  | 3 | exp Health Education/ | 16687 | Advanced | [Display Results](http://ovidsp.uk.ovid.com/sp-3.25.0a/ovidweb.cgi?&S=FMDJPDABLJHFJNEKFNGKCAJHEDGOAA00&SELECT=S.sh%7c&R=3&Process+Action=display)  [More](http://ovidsp.uk.ovid.com/sp-3.25.0a/ovidweb.cgi) |  |
|  | 4 | weight.mp. or exp WEIGHT LOSS/ | 69770 | Advanced | [Display Results](http://ovidsp.uk.ovid.com/sp-3.25.0a/ovidweb.cgi?&S=FMDJPDABLJHFJNEKFNGKCAJHEDGOAA00&SELECT=S.sh%7c&R=4&Process+Action=display)  [More](http://ovidsp.uk.ovid.com/sp-3.25.0a/ovidweb.cgi) |  |
|  | 5 | physical activity.mp. or exp Physical Activity/ | 44098 | Advanced | [Display Results](http://ovidsp.uk.ovid.com/sp-3.25.0a/ovidweb.cgi?&S=FMDJPDABLJHFJNEKFNGKCAJHEDGOAA00&SELECT=S.sh%7c&R=5&Process+Action=display)  [More](http://ovidsp.uk.ovid.com/sp-3.25.0a/ovidweb.cgi) |  |
|  | 6 | exp EXERCISE/ or exercise.mp. | 49918 | Advanced | [Display Results](http://ovidsp.uk.ovid.com/sp-3.25.0a/ovidweb.cgi?&S=FMDJPDABLJHFJNEKFNGKCAJHEDGOAA00&SELECT=S.sh%7c&R=6&Process+Action=display)  [More](http://ovidsp.uk.ovid.com/sp-3.25.0a/ovidweb.cgi) |  |
|  | 7 | exp SMOKING CESSATION/ or smoking.mp. | 47030 | Advanced | [Display Results](http://ovidsp.uk.ovid.com/sp-3.25.0a/ovidweb.cgi?&S=FMDJPDABLJHFJNEKFNGKCAJHEDGOAA00&SELECT=S.sh%7c&R=7&Process+Action=display)  [More](http://ovidsp.uk.ovid.com/sp-3.25.0a/ovidweb.cgi) |  |
|  | 8 | exp Alcohol Drinking Patterns/ or alcohol intake.mp. | 61892 | Advanced | [Display Results](http://ovidsp.uk.ovid.com/sp-3.25.0a/ovidweb.cgi?&S=FMDJPDABLJHFJNEKFNGKCAJHEDGOAA00&SELECT=S.sh%7c&R=8&Process+Action=display)  [More](http://ovidsp.uk.ovid.com/sp-3.25.0a/ovidweb.cgi) |  |
|  | 9 | barriers.mp. | 45426 | Advanced | [Display Results](http://ovidsp.uk.ovid.com/sp-3.25.0a/ovidweb.cgi?&S=FMDJPDABLJHFJNEKFNGKCAJHEDGOAA00&SELECT=S.sh%7c&R=9&Process+Action=display)  [More](http://ovidsp.uk.ovid.com/sp-3.25.0a/ovidweb.cgi) |  |
|  | 10 | facilitators.mp. | 6615 | Advanced | [Display Results](http://ovidsp.uk.ovid.com/sp-3.25.0a/ovidweb.cgi?&S=FMDJPDABLJHFJNEKFNGKCAJHEDGOAA00&SELECT=S.sh%7c&R=10&Process+Action=display)  [More](http://ovidsp.uk.ovid.com/sp-3.25.0a/ovidweb.cgi) |  |
|  | 11 | beliefs.mp. | 91780 | Advanced | [Display Results](http://ovidsp.uk.ovid.com/sp-3.25.0a/ovidweb.cgi?&S=FMDJPDABLJHFJNEKFNGKCAJHEDGOAA00&SELECT=S.sh%7c&R=11&Process+Action=display)  [More](http://ovidsp.uk.ovid.com/sp-3.25.0a/ovidweb.cgi) |  |
|  | 12 | exp Health Personnel Attitudes/ | 20563 | Advanced | [Display Results](http://ovidsp.uk.ovid.com/sp-3.25.0a/ovidweb.cgi?&S=FMDJPDABLJHFJNEKFNGKCAJHEDGOAA00&SELECT=S.sh%7c&R=12&Process+Action=display)  [More](http://ovidsp.uk.ovid.com/sp-3.25.0a/ovidweb.cgi) |  |
|  | 13 | opinions.mp. | 20904 | Advanced | [Display Results](http://ovidsp.uk.ovid.com/sp-3.25.0a/ovidweb.cgi?&S=FMDJPDABLJHFJNEKFNGKCAJHEDGOAA00&SELECT=S.sh%7c&R=13&Process+Action=display)  [More](http://ovidsp.uk.ovid.com/sp-3.25.0a/ovidweb.cgi) |  |
|  | 14 | perceptions.mp. | 136505 | Advanced | [Display Results](http://ovidsp.uk.ovid.com/sp-3.25.0a/ovidweb.cgi?&S=FMDJPDABLJHFJNEKFNGKCAJHEDGOAA00&SELECT=S.sh%7c&R=14&Process+Action=display)  [More](http://ovidsp.uk.ovid.com/sp-3.25.0a/ovidweb.cgi) |  |
|  | 15 | exp Meta Analysis/ | 4002 | Advanced | [Display Results](http://ovidsp.uk.ovid.com/sp-3.25.0a/ovidweb.cgi?&S=FMDJPDABLJHFJNEKFNGKCAJHEDGOAA00&SELECT=S.sh%7c&R=15&Process+Action=display)  [More](http://ovidsp.uk.ovid.com/sp-3.25.0a/ovidweb.cgi) |  |
|  | 16 | synthesis.mp. | 27353 | Advanced | [Display Results](http://ovidsp.uk.ovid.com/sp-3.25.0a/ovidweb.cgi?&S=FMDJPDABLJHFJNEKFNGKCAJHEDGOAA00&SELECT=S.sh%7c&R=16&Process+Action=display)  [More](http://ovidsp.uk.ovid.com/sp-3.25.0a/ovidweb.cgi) |  |
|  | 17 | exp "LITERATURE REVIEW"/ or review.mp. | 332544 | Advanced | [Display Results](http://ovidsp.uk.ovid.com/sp-3.25.0a/ovidweb.cgi?&S=FMDJPDABLJHFJNEKFNGKCAJHEDGOAA00&SELECT=S.sh%7c&R=17&Process+Action=display)  [More](http://ovidsp.uk.ovid.com/sp-3.25.0a/ovidweb.cgi) |  |
|  | 18 | 2 or 3 | 35648 | Advanced | [Display Results](http://ovidsp.uk.ovid.com/sp-3.25.0a/ovidweb.cgi?&S=FMDJPDABLJHFJNEKFNGKCAJHEDGOAA00&SELECT=S.sh%7c&R=18&Process+Action=display)  [More](http://ovidsp.uk.ovid.com/sp-3.25.0a/ovidweb.cgi) |  |
|  | 19 | 4 or 5 or 6 or 7 or 8 | 227244 | Advanced | [Display Results](http://ovidsp.uk.ovid.com/sp-3.25.0a/ovidweb.cgi?&S=FMDJPDABLJHFJNEKFNGKCAJHEDGOAA00&SELECT=S.sh%7c&R=19&Process+Action=display)  [More](http://ovidsp.uk.ovid.com/sp-3.25.0a/ovidweb.cgi) |  |
|  | 20 | 9 or 10 or 11 or 12 or 13 or 14 | 289677 | Advanced | [Display Results](http://ovidsp.uk.ovid.com/sp-3.25.0a/ovidweb.cgi?&S=FMDJPDABLJHFJNEKFNGKCAJHEDGOAA00&SELECT=S.sh%7c&R=20&Process+Action=display)  [More](http://ovidsp.uk.ovid.com/sp-3.25.0a/ovidweb.cgi) |  |
|  | 21 | 15 or 16 or 17 | 356382 | Advanced | [Display Results](http://ovidsp.uk.ovid.com/sp-3.25.0a/ovidweb.cgi?&S=FMDJPDABLJHFJNEKFNGKCAJHEDGOAA00&SELECT=S.sh%7c&R=21&Process+Action=display)  [More](http://ovidsp.uk.ovid.com/sp-3.25.0a/ovidweb.cgi) |  |
|  | 22 | 1 and 18 and 19 and 20 and 21 | 7 | Advanced | [Display Results](http://ovidsp.uk.ovid.com/sp-3.25.0a/ovidweb.cgi?&S=FMDJPDABLJHFJNEKFNGKCAJHEDGOAA00&SELECT=S.sh%7c&R=22&Process+Action=display)  [More](http://ovidsp.uk.ovid.com/sp-3.25.0a/ovidweb.cgi) |  |

**Sportdiscus:**

(“Health professional” OR “Health practitioner” OR “Health personnel” OR Anaesthetist OR Audiologist OR Chiropractor OR Dentist OR Dietician OR Dietitian OR “General practitioner” OR Doctor OR Gynaecologist OR “Health visitor” OR Midwife OR Nurse OR Obstetrician OR Ophthalmologist OR Optician OR Osteopath OR Paediatrician OR Pathologist OR Pharmacist OR Psychiatrist OR Psychologist OR Radiologist OR “Social Worker” OR “Speech therapist” OR “Language therapist” OR Surgeon OR Physician OR “Primary care” OR “Secondary care” OR pediatrician ) AND ( “Health Information” OR “Health Advice” OR “Health Intervention” OR “Service provision” OR “Behavio?r change” OR “lifestyle intervention” OR Counsel?ing OR “Health Communication” OR “Health Promotion” OR “Health education” ) AND (Diet OR Nutrition OR Fruit OR Vegetables OR Sugar OR Fat OR Fibre OR Salt OR “Physical activity” OR Exercise OR Inactivity OR “Weight loss” OR BMI OR “Body mass index” OR “Sedentary behavio?r”  OR smoking OR alcohol) AND barriers OR facilitators OR beliefs OR attitudes OR opinions OR perceptions AND AB (Review OR Meta-analysis OR synthesis )

**Cochrane library:**

Search Name:

Date Run: 02/06/17 10:32:08.711

Description:

ID Search Hits

#1 MeSH descriptor: [Health Personnel] explode all trees 7623

#2 MeSH descriptor: [Health Promotion] explode all trees 5487

#3 MeSH descriptor: [Health Education] explode all trees 12125

#4 MeSH descriptor: [Health Communication] explode all trees 108

#5 MeSH descriptor: [Life Style] explode all trees 4040

#6 MeSH descriptor: [Alcohol Drinking] explode all trees 3017

#7 MeSH descriptor: [Smoking Cessation] explode all trees 3812

#8 barriers:ti,ab,kw (Word variations have been searched) 8261

#9 facilitators:ti,ab,kw (Word variations have been searched) 1009

#10 beliefs:ti,ab,kw (Word variations have been searched) 4218

#11 MeSH descriptor: [Attitude of Health Personnel] explode all trees 2095

#12 opinions:ti,ab,kw (Word variations have been searched) 3400

#13 perceptions:ti,ab,kw (Word variations have been searched) 18961

#14 #2 or #3 or #4 16616

#15 #5 or #6 or #7 10628

#16 #8 or #9 or #10 or #11 or #12 or #13 35212

#17 #1 and #14 and #15 and #16 21
