# Supplementary material for: Delivering Opportunistic Behavior Change Interventions: a Systematic Review of Systematic Reviews
Source: Prev Sci. 2020 Feb 17;21(3):319–31. doi: 10.1007/s11121-020-01087-6 (PMC7056685; doi:10.1007/s11121-020-01087-6)
Supplement: Supplementary file 2 — (DOCX 105 kb) [file 11121_2020_1087_MOESM2_ESM.docx]

**Supplementary File C.** Full reference list of included reviews

Alonso-Perales, M. D., Lasheras, B., Beitia, G., Beltran, I., Marcos, B., & Nunez-Cordoba, J. M. (2017). Barriers to promote cardiovascular health in community pharmacies: a systematic review. *Health Promotion International, 32*(3), 535-548. doi:10.1093/heapro/dav098

Anderson, C., Blenkinsopp, A., & Armstrong, M. (2003). Pharmacists' perceptions regarding their contribution to improving the public's health: a systematic review of the United Kingdom and international literature 1990–2001. *International Journal of Pharmacy Practice, 11*(2), 111-120. doi:10.1211/0022357021297

Bakhshi, S., & While, A. E. (2014). Health professionals' alcohol-related professional practices and the relationship between their personal alcohol attitudes and behavior and professional practices: A systematic review. *International Journal of Environmental Research and Public Health, 11*(1), 218-248. doi:<http://dx.doi.org/10.3390/ijerph110100218>

Baxter, S., Everson-Hock, E., Messina, J., Guillaume, L., Burrows, J., & Goyder, E. (2010). Factors relating to the uptake of interventions for smoking cessation among pregnant women: a systematic review and qualitative synthesis. *Nicotine Tob Res, 12*(7), 685-694. doi:10.1093/ntr/ntq072

Bock, C., Diehl, K., Schneider, S., Diehm, C., & Litaker, D. (2012). Behavioral Counseling for Cardiovascular Disease Prevention in Primary Care Settings: A Systematic Review of Practice and Associated Factors. *Medical Care Research and Review, 69*(5), 495-518. doi:10.1177/1077558712441084

Conlon, K., Pattinson, L., & Hutton, D. (2017). Attitudes of oncology healthcare practitioners towards smoking cessation: A systematic review of the facilitators, barriers and recommendations for delivery of advice and support to cancer patients. *Radiography (Lond), 23*(3), 256-263. doi:10.1016/j.radi.2017.03.006

Cooley, M. E., Lundin, R., & Murray, L. (2009). Smoking cessation interventions in cancer care: opportunities for oncology nurses and nurse scientists. *Annu Rev Nurs Res, 27*, 243-272.

Crisford, P., Winzenberg, T., Venn, A., Schultz, M., Aitken, D., & Cleland, V. (2018). Factors associated with physical activity promotion by allied and other non-medical health professionals: A systematic review. *Patient Educ Couns, 101*(10), 1775-1785. doi:10.1016/j.pec.2018.05.011

Dewhurst, A., Peters, S., Devereux-Fitzgerald, A., & Hart, J. (2017). Physicians' views and experiences of discussing weight management within routine clinical consultations: A thematic synthesis. *Patient Education and Counseling, 100*(5), 897-908. doi:10.1016/j.pec.2016.12.017

Duaso, M. J., McDermott, M. S., Mujika, A., Purssell, E., & While, A. (2014). Do doctors' smoking habits influence their smoking cessation practices? A systematic review and meta-analysis. *Addiction, 109*(11), 1811-1823. doi:10.1111/add.12680

Eakin, E. G., Smith, B. J., & Bauman, A. E. (2005). Evaluating the Population Health Impact of Physical Activity Interventions in Primary Care--Are We Asking the Right Questions? *Journal of Physical Activity & Health, 2*(2), 197.

Fie, S., Norman, I. J., & While, A. E. (2013). The relationship between physicians’ and nurses’ personal physical activity habits and their health-promotion practice: A systematic review. *Health Education Journal, 72*(1), 102-119.

Flemming, K., Graham, H., McCaughan, D., Angus, K., Sinclair, L., & Bauld, L. (2016). Health professionals' perceptions of the barriers and facilitators to providing smoking cessation advice to women in pregnancy and during the post-partum period: a systematic review of qualitative research. *BMC Public Health, 16*, 290. doi:10.1186/s12889-016-2961-9

Gentry, S., Craig, J., Holland, R., & Notley, C. (2017). Smoking cessation for substance misusers: A systematic review of qualitative studies on participant and provider beliefs and perceptions. *Drug Alcohol Depend, 180*, 178-192. doi:10.1016/j.drugalcdep.2017.07.043

Guydish, J., Passalacqua, E., Tajima, B., & Manser, S. T. (2007). Staff smoking and other barriers to nicotine dependence intervention in addiction treatment settings: a review. *J Psychoactive Drugs, 39*(4), 423-433. doi:10.1080/02791072.2007.10399881

Hebert, E. T., Caughy, M. O., & Shuval, K. (2012). Primary care providers' perceptions of physical activity counselling in a clinical setting: a systematic review. *British Journal of Sports Medicine, 46*(9), 625-631.

Heslehurst, N., Newham, J., Maniatopoulos, G., Fleetwood, C., Robalino, S., & Rankin, J. (2014). Implementation of pregnancy weight management and obesity guidelines: a meta-synthesis of healthcare professionals' barriers and facilitators using the Theoretical Domains Framework. *Obes Rev, 15*(6), 462-486. doi:10.1111/obr.12160

Huijg, J., Gebhardt, W., Verheijden, M., Zouwe, N., Vries, J., Middelkoop, B., & Crone, M. (2015). Factors Influencing Primary Health Care Professionals' Physical Activity Promotion Behaviors: A Systematic Review. *International Journal of Behavioral Medicine, 22*(1), 32-50.

Johnson, M., Jackson, R., Guillaume, L., Meier, P., & Goyder, E. (2011). Barriers and facilitators to implementing screening and brief intervention for alcohol misuse: a systematic review of qualitative evidence. *Journal of Public Health, 33*(3), 412-421. doi:pubmed/fdq095

Kelly, M., Wills, J., & Sykes, S. (2017). Do nurses' personal health behaviours impact on their health promotion practice? A systematic review. *International Journal of Nursing Studies, 76*, 62-77. doi:10.1016/j.ijnurstu.2017.08.008

Knudsen, H. K. (2017). Implementation of smoking cessation treatment in substance use disorder treatment settings: a review. *Am J Drug Alcohol Abuse, 43*(2), 215-225. doi:10.1080/00952990.2016.1183019

Lala, R., Csikar, J., Douglas, G., & Muarry, J. (2017). Factors that influence delivery of tobacco cessation support in general dental practice: a narrative review. *Journal of Public Health Dentistry, 77*(1), 47-53. doi:10.1111/jphd.12170

Lucas, C., Charlton, K. E., & Yeatman, H. (2014). Nutrition advice during pregnancy: do women receive it and can health professionals provide it? *Matern Child Health J, 18*(10), 2465-2478. doi:10.1007/s10995-014-1485-0

Oxman, A. D., Thomson, M. A., Davis, D. A., & Haynes, R. B. (1995). No magin bullets – A systematic review of102 trials of interventions to improve professional practice. *Canadian Medical Association Journal, 153*(10), 1423-1431.

Rosseel, J. P., Jacobs, J. E., Plasschaert, A. J. M., & Grol, R. P. T. M. (2012). A review of strategies to stimulate dental professionals to integrate smoking cessation interventions into primary care. *Community Dental Health, 29*(2), 154-161. doi:10.1922/CDH_2595Rosseel08

Stead, M., Angus, K., Holme, I., Cohen, D., Tait, G., Peña, C. C., . . . Costa, J. (2009). Factors influencing European GPs' engagement in smoking cessation: A multi-country literature review. *British Journal of General Practice, 59*(566), 682-690. doi:10.3399/bjgp09X454007

Teixeira, F. V., Pais-Ribeiro, J. L., & Maia, A. R. (2012). Beliefs and practices of healthcare providers regarding obesity: a systematic review. *Rev Assoc Med Bras (1992), 58*(2), 254-262.

Thompson, M., Robertson, J., & Clough, A. (2011). A review of the barriers preventing Indigenous Health Workers delivering tobacco interventions to their communities. *Aust N Z J Public Health, 35*(1), 47-53. doi:10.1111/j.1753-6405.2010.00632.x

Van Gerwen, M., Franc, C., Rosman, S., Le Vaillant, M., & Pelletier-Fleury, N. (2009). Primary care physicians' knowledge, attitudes, beliefs and practices regarding childhood obesity: A systematic review: Obesity Review. *Obesity Reviews, 10*(2), 227-236. doi:<http://dx.doi.org/10.1111/j.1467-789X.2008.00532.x>

Verhaeghe, N., De Maeseneer, J., Maes, L., Van Heeringen, C., & Annemans, L. (2011). Perceptions of mental health nurses and patients about health promotion in mental health care: a literature review. *J Psychiatr Ment Health Nurs, 18*(6), 487-492. doi:10.1111/j.1365-2850.2011.01692.x

Vine, M., Hargreaves, M. B., Briefel, R. R., & Orfield, C. (2013). Expanding the role of primary care in the prevention and treatment of childhood obesity: A review of clinic- and community-based recommendations and interventions. *Journal of Obesity, 2013*. doi:10.1155/2013/172035

Vogt, F., Hall, S., & Marteau, T. M. (2005). General practitioners' and family physicians' negative beliefs and attitudes towards discussing smoking cessation with patients: A systematic review. *Addiction, 100*(10), 1423-1431. doi:<http://dx.doi.org/10.1111/j.1360-0443.2005.01221.x>

Wandell, P. E., de Waard, A. K. M., Holzmann, M. J., Gornitzki, C., Lionis, C., de Wit, N., . . . Carlsson, A. C. (2018). Barriers and facilitators among health professionals in primary care to prevention of cardiometabolic diseases: A systematic review. *Family Practice, 35*(4), 383-398. doi:10.1093/fampra/cmx137

Yousefzadeh, A., Chung, F., Wong, D. T., Warner, D. O., & Wong, J. (2016). Smoking Cessation: The Role of the Anesthesiologist. *Anesthesia and Analgesia, 122*(5), 1311-1320. doi:10.1213/ane.0000000000001170

Zhu, D., Norman, I. J., & While, A. E. (2011). The relationship between health professionals' weight status and attitudes towards weight management: a systematic review. *Obes Rev, 12*(5), e324-337. doi:10.1111/j.1467-789X.2010.00841.x
