# Supplementary material for: Delivering Opportunistic Behavior Change Interventions: a Systematic Review of Systematic Reviews
Source: Prev Sci. 2020 Feb 17;21(3):319–31. doi: 10.1007/s11121-020-01087-6 (PMC7056685; doi:10.1007/s11121-020-01087-6)
Supplement: Supplementary file 3 — (DOCX 106 kb) [file 11121_2020_1087_MOESM3_ESM.docx]

**Supplementary File C.** Review characteristics

| **Author and year** | **Review type** | **Quantitative/ Qualitative studies** | **Setting (inc. whether part of inclusion criteria)** | **Type of healthcare professional** | **No. of studies** | **Included studies (design/data collection type)** | **Study quality** | **Coded themes** |
| --- | --- | --- | --- | --- | --- | --- | --- | --- |
| Anderson 2003 | Systematic review | Qualitative and quantitative | Not reported | Pharmacists (4), community pharmacists (8) | 12 | Semi-structured interviews (1), in-depth interviews (3), pre- and post-training interviews (1), structured interviews (3), cross sectional survey with telephone interview follow-up (1), cross-sectional survey (3) | 5/9 | - Perceived lack of prioritisation of health behaviour change as a clinical priority - Training as an enabler to delivering behaviour change interventions - Contextual enablers - Perceptions of the healthcare professional role (enabler) |
| Alonso-Perales 2017 | Systematic review | Quantitative | Community pharmacy | Community pharmacists | 24 | Cross-sectional surveys | 5/9 | - Perceptions of the knowledge or skills needed to support behaviour change with patients (barrier) - Perceived lack of time to deliver behaviour change interventions |
| Bakhshi 2014 | Systematic review | Qualitative and quantitative | Not reported | Doctors (2), nurses (2), dentists (1), multiple hcps (1) | 6 (in strand 2) | Cross-sectional survey (5), quasi experimental study (1) | 5/9 | - Attitudes towards delivering behaviour change interventions |
| Baxter 2010 | Systematic review and qualitative synthesis | Qualitative, quantitative, and narrative description from papers reporting RCTs | Not reported | Not reported | 23 | No restrictions on study design  10 qualitative, 10 cross-sectional surveys, three were narrative descriptions related to papers reported RCTs | 6/9 | - Beliefs about resources and support needed to facilitate intervention delivery (barrier) - Perceptions of the knowledge or skills needed to support behaviour change with patients (barrier) - Perceptions of the knowledge or skills needed to support behaviour change with patients (enabler) - Perceptions of the healthcare professional role (barrier) - Perceived lack of time to deliver behaviour change interventions - Attitudes towards patients and perceptions of patient risk prevents delivery of interventions |
| Bock 2012 | Systematic review | Quantitative (observational studies or self-report surveys) | Primary care | Primary care physicians (2), GPs (5), resident physicians (2), family physicians (1) multiple hcps (8) | 18 unique studies - 21 articles | Exact numbers not reported | 5/9 | - Perceptions of the healthcare professional role (enabler) - Attitudes towards patients and perceptions of patient risk prevents delivery of interventions - Perceptions of the knowledge or skills needed to support behaviour change with patients (barrier) |
| Cooley 2009 | Systematic review | Quantitative | Not stated | Nurses (7), physician/nurse practitioner (3), health educator (1), physicians/dentist (1), counsellor (1), physicians (2), tobacco treatment specialists (2), hcps (1) | 21 (18 with hcps) | RCT (13), quasi-experimental (8) | 3/9 | - Attitudes towards delivering behaviour change interventions - Perceptions of the knowledge or skills needed to support behaviour change with patients (barrier) |
| Conlon 2017 | Systematic review | Qualitative and quantitative | Not reported | Oncology nurses (3), Nurses (3), Doctors (3), Multiple hcps (5), Otolaryngologists (1), Complementary Therapists (1), Therapeutic Radiographers (1), Thoracic oncology HCPs (1) | 17 | Survey (12), diary (1), other (1), interviews (1), survey and focus groups (3), | 4/9 | - Perceptions of the healthcare professional role (barrier) - Healthcare professionals’ own health behaviour (barrier) - Perceived lack of time to deliver behaviour change interventions - Healthcare professionals’ perceptions of patient motivation - Training as an enabler to delivering behaviour change interventions - Attitudes towards delivering behaviour change interventions - Perceptions of the knowledge or skills needed to support behaviour change with patients (barrier) |
| Crisford 2018 | Systematic review | Quantitative | Multiple settings (16), inpatient and outpatient (1), community primary healthcare (3), hospital (2), not reported (4), general practice (2), private practice (1), inpatient mental health facilities (1) | Dieticians (3), dental hygienists (1),  health care support workers (1), nurses (9), practice nurses  (2), nurse practitioners (4), nurse midwives (1), oncology  nurses (2), psychiatric nurses (1), occupational therapists  (1), practice assistants (1), physiotherapists (9), psychologists/  Psychotherapists (4), mixed allied health practitioners  (2). | 30 | Surveys | 7/9 | - Training as an enabler to delivering behaviour change interventions - Perceptions of the knowledge or skills needed to support behaviour change with patients (enabler) |
| Dewhurst 2017 | Thematic synthesis | Qualitative | Primary care? Not part of inclusion | Physicians | 16 | Semi-structured interviews (11), in-depth interviews (1), focus groups (3), focus groups plus semi-structured interviews (1) | 4/9 | - Beliefs about resources and support needed to facilitate intervention delivery (barrier) - Perceived lack of time to deliver behaviour change interventions - Perceived lack of prioritisation of health behaviour change as a clinical priority - Attitudes towards patients and perceptions of patient risk prevents delivery of interventions - Healthcare professionals’ perceptions of patient motivation - Perceptions of the healthcare professional role (enabler) |
| Duaso 2014 | Systematic review and meta-analysis | Quantitative | Not reported | Cardiologist (1), Hospital physician (3), non-primary care women physicians (1), primary care women physician (1), doctors specialised in infertility (1), primary care physicians (1), physicians (2), GPs (2), physician with primary clinical responsibilities (1), non-vascular surgeons (1), hospital doctors (1), physicians not specified (1), oncologists (1), physicians from all specialities (1), GPs and general internists (1), physicians (faculty and health service) (1) | 20 | Surveys | 7/11 | - Healthcare professionals’ own health behaviour (barrier) |
| Eakin 2005 | Systematic review | Quantitative | Primary care (part of inclusion) | Physicians | 8 | Physician self-reported questionnaires | 3/9 | - Perceived lack of time to deliver behaviour change interventions - Perceived lack of prioritisation of health behaviour change as a clinical priority - Attitudes towards patients and perceptions of patient risk prevents delivery of interventions - Healthcare professionals’ perceptions of patient motivation - Perceptions of the knowledge or skills needed to support behaviour change with patients (barrier) |
| Fie 2011 | Systematic review | Quantitative | Not reported | Nurses (2), multiple hcps (3), physicians/GPs (5), Oncologist (1), not reported (1), internal medicine residents (1) | 13 | Cross-sectional surveys | 5/9 | - Attitudes towards delivering behaviour change interventions - Healthcare professionals’ own health behaviour (enabler) |
| Flemming 2016 | Systematic review of qualitative studies | Qualitative | Not reported | Midwives (4), multiple hcps (4) | 8 studies, 9 papers | Focus groups and interviews | 5/9 | - Perceptions of the knowledge or skills needed to support behaviour change with patients (enabler) - Contextual enablers |
| Gentry 2017 | Systematic review | Qualitative | Not reported | Treatment providers (5), counsellors and clinical supervisors (1), staff in substance misuse facilities (2) | 8 studies with HCP focus (3 hcp only and 5 hcp and service users) | Open ended survey items (3), interviews (5) | 6/9 | - Perceptions of the healthcare professional role (barrier) - Beliefs about resources and support needed to facilitate intervention delivery (barrier) - Healthcare professionals’ own health behaviour (barrier) - Contextual enablers - Perceptions of the knowledge or skills needed to support behaviour change with patients (barrier) |
| Guydish 2007 | Systematic review | Qualitative and quantitative | Not reported | Not reported | 20 | Interviews and self-report surveys | 2/9 | - Beliefs about resources and support needed to facilitate intervention delivery (barrier) - Healthcare professionals’ own health behaviour (barrier) - Perceived lack of time to deliver behaviour change interventions - Perceived lack of prioritisation of health behaviour change as a clinical priority - Attitudes towards patients and perceptions of patient risk prevents delivery of interventions - Healthcare professionals’ perceptions of patient motivation - Perceptions of the knowledge or skills needed to support behaviour change with patients (barrier) |
| Hebert 2011 | Systematic review | Qualitative and quantitative | Primary care (part of inclusion) | Nurses (7), physicians (12). Check table | 19 | Cross-sectional surveys and 1 qualitative, 2 mixed methods | 3/9 | - Beliefs about resources and support needed to facilitate intervention delivery (barrier) - Healthcare professionals’ own health behaviour (enabler) - Perceived lack of time to deliver behaviour change interventions - Perceived lack of prioritisation of health behaviour change as a clinical priority - Attitudes towards patients and perceptions of patient risk prevents delivery of interventions - Perceptions of the knowledge or skills needed to support behaviour change with patients (barrier) |
| Heslehurst 2014 | Systematic review - meta-synthesis | Qualitative and quantitative | Not reported | Multiple hcps (14), midwives (5), Obstetricians/gynaecologists (3), nurses (2), GP (1) | 25 | Interviews, focus groups observations, questionnaires | 5/9 | - Perceptions of the knowledge or skills needed to support behaviour change with patients (barrier) - Perceptions of the knowledge or skills needed to support behaviour change with patients (enabler) - Perceptions of the healthcare professional role (barrier) - Perceptions of the healthcare professional role (enabler) - Beliefs about resources and support needed to facilitate intervention delivery (barrier) - Healthcare professionals’ own health behaviour (barrier) - Perceived lack of time to deliver behaviour change interventions - Perceived lack of prioritisation of health behaviour change as a clinical priority - Attitudes towards patients and perceptions of patient risk prevents delivery of interventions - Healthcare professionals’ perceptions of patient motivation - Training as an enabler to delivering behaviour change interventions |
| Hujig 2015 | Systematic literature review | Qualitative and quantitative | Primary health care | Multiple hcps (5), PHC professionals (11), Physician/GP (23), not reported (13), Nurses (3), Dieticians (2) | 59 | Questionnaires, interviews, Focus groups, | 3/9 | - Perceptions of the knowledge or skills needed to support behaviour change with patients (barrier) - Beliefs about resources and support needed to facilitate intervention delivery (barrier) - Beliefs about resources and support needed to facilitate intervention delivery (enabler) - Perceived lack of time to deliver behaviour change interventions - Perceived lack of prioritisation of health behaviour change as a clinical priority - Attitudes towards patients and perceptions of patient risk prevents delivery of interventions - Healthcare professionals’ perceptions of patient motivation - Contextual enablers - Attitudes towards delivering behaviour change interventions |
| Johnson 2010 | Systematic review of qualitative evidence | Qualitative and quantitative (e.g. RCTs which included a discussion about implementation) | Primary care, emergency care, secondary care, and probation centres (mostly PC) | GPs (13), Nurses (5), forensic medical examiners (1), receptionists (1), Surgeons (1), multiple hcps (5), not reported (17) | 47 | Reviews, RCTs, surveys and qualitative studies | 4/9 | - Perceptions of the healthcare professional role (barrier) - Perceptions of the healthcare professional role (enabler) - Perceived lack of prioritisation of health behaviour change as a clinical priority - Training as an enabler to delivering behaviour change interventions - Contextual enablers - Perceptions of the knowledge or skills needed to support behaviour change with patients (barrier) |
| Kelly 2017 | Systematic review | Qualitative and quantitative | Primary care (4), hospitals (13), community care (1), multiple settings (11) | Nurses | 29 | Cross-sectional surveys (22), Qualitative (6), mixed methods (1) | 5/9 | - Beliefs about resources and support needed to facilitate intervention delivery (enabler) - Healthcare professionals’ own health behaviour (barrier) - Training as an enabler to delivering behaviour change interventions - Beliefs about resources and support needed to facilitate intervention delivery (barrier) |
| Knudsen 2017 | Systematic review | Qualitative and quantitative | Specialty SUD treatment  settings | Not reported | 51 | Not reported | 0/9 | - Perceived lack of prioritisation of health behaviour change as a clinical priority - Beliefs about resources and support needed to facilitate intervention delivery (barrier) - Perceptions of the knowledge or skills needed to support behaviour change with patients (barrier) |
| Lala 2016 | Systematic narrative review | Quantitative | Primary care general dental practice | Dentists | 6 | Observational | 5/9 | - Healthcare professionals’ own health behaviour (barrier) - Contextual enablers - Beliefs about resources and support needed to facilitate intervention delivery (enabler) |
| Lucas 2014 | Systematic literature review | Qualitative and quantitative | Not reported | Midwives (1), multiple hcps (5) | 25 (6 focused on hcps) | Observational studies | 4/9 | - Perceptions of the knowledge or skills needed to support behaviour change with patients (enabler) - Beliefs about resources and support needed to facilitate intervention delivery (barrier) - Perceived lack of time to deliver behaviour change interventions - Perceived lack of prioritisation of health behaviour change as a clinical priority - Perceptions of the knowledge or skills needed to support behaviour change with patients (barrier) |
| Oxman 1995 | Systematic review | Quantitative (trials) | Not reported | Not reported | 102trials | Trials | 4/9 | - Beliefs about resources and support needed to facilitate intervention delivery (enabler) - Perceptions of the healthcare professional role (enabler) |
| Rosseel 2012 | Systematic review | Controlled studies and systematic reviews | Primary care general dental practice | Dental professionals | 8 | RCTs | 5/9 | - Training as an enabler to delivering behaviour change interventions - Beliefs about resources and support needed to facilitate intervention delivery (enabler) |
| Stead 2009 | Literature review using systematic search | Qualitative and quantitative | Primary acre (presumed as GPs) | GPs | 100 | Not reported (only qual and quant stated) | 3/9 | - Perceptions of the knowledge or skills needed to support behaviour change with patients (barrier) - Perceptions of the healthcare professional role (barrier) - Perceptions of the healthcare professional role (enabler) - Beliefs about resources and support needed to facilitate intervention delivery (barrier) - Healthcare professionals’ own health behaviour (barrier) - Perceived lack of time to deliver behaviour change interventions - Perceived lack of prioritisation of health behaviour change as a clinical priority - Attitudes towards patients and perceptions of patient risk prevents delivery of interventions - Healthcare professionals’ perceptions of patient motivation - Training as an enabler to delivering behaviour change interventions - Healthcare professionals’ own health behaviour (enabler) |
| Teixeira 2011 | Systematic review | Qualitative and quantitative | Primary care | General and family practitioners | 13 | Focus group (1), questionnaire based on case reports (2), Questionnaire (9), Interviews (1) | 2/9 | - Perceptions of the healthcare professional role (barrier) - Perceptions of the healthcare professional role (enabler) - Perceived lack of time to deliver behaviour change interventions - Attitudes towards patients and perceptions of patient risk prevents delivery of interventions - Healthcare professionals’ perceptions of patient motivation - Perceptions of the knowledge or skills needed to support behaviour change with patients (barrier) |
| Thompson 2011 | Literature review using systematic search | Qualitative and quantitative | Not reported | Health workers | 14 | Report (1), opinion (1), unpublished (2), follow-up study (3), cross-sectional study (7) | 3/9 | - Healthcare professionals’ own health behaviour (barrier) - Perceived lack of prioritisation of health behaviour change as a clinical priority - Training as an enabler to delivering behaviour change interventions - Perceptions of the healthcare professional role (barrier) |
| van Dillen 2014 | Systematic review | Qualitative and quantitative | Primary care | Nurses (15), multiple hcps (30) | 45 | Questionnaires, observations, focus groups. Interviews | 4/9 | - Perceptions of the healthcare professional role (barrier) - Perceived lack of time to deliver behaviour change interventions - Perceived lack of prioritisation of health behaviour change as a clinical priority - Perceptions of the healthcare professional role (barrier) |
| van Gerwen 2008 | Systematic review | Quantitative | Primary care | Paediatricians (8), Multiple hcps (2), PC physician (1), GP (1) | 11 | Cross-sectional questionnaires | 3/9 | - Attitudes towards delivering behaviour change interventions - Beliefs about resources and support needed to facilitate intervention delivery (enabler) |
| Verhareghe  2011 | Literature review using systematic search | Qualitative | In- and out-patient mental health care | Mental health nurses | 14 (4 were based on mental health nurses) | Interviews and focus groups (related to the 4 studies focused on mh nurses) | 2/9 | - Perceived lack of prioritisation of health behaviour change as a clinical priority - Perceptions of the healthcare professional role (barrier) |
| Vine 2013 | Literature review using systematic search | Qualitative and quantitative | Primary care, or community care with a direct link to primary care | Primary care providers | 96 | 63 articles describing specific interventions, 14 reviewed existing interventions, 13 summarising recommendations for the treatment and prevention of childhood obesity, and 6 summarising the results of topic-related focus groups with parents, children or clinicians | 1/9 | - Perceptions of the healthcare professional role (barrier) - Beliefs about resources and support needed to facilitate intervention delivery (barrier) - Beliefs about resources and support needed to facilitate intervention delivery (enabler) - Perceived lack of time to deliver behaviour change interventions - Training as an enabler to delivering behaviour change interventions - Contextual enablers - Perceptions of the healthcare professional role (enabler) - Perceptions of the knowledge or skills needed to support behaviour change with patients (enabler) |
| Vogt 2005 | Systematic review | Quantitative | Primary care (not explicitly stated) | GPs and family physicians | 20 | Survey (18), intervention (2) | 2/9 | - Perceived lack of time to deliver behaviour change interventions - Perceived lack of prioritisation of health behaviour change as a clinical priority - Attitudes towards patients and perceptions of patient risk prevents delivery of interventions - Perceptions of the healthcare professional role (barrier) - Perceptions of the knowledge or skills needed to support behaviour change with patients (barrier) |
| Wandell 2018 | Systematic review | Qualitative and quantitative | Primary care | GPs (13), multiple (13), CDPM programme delivery staff (1) | 27 | Interviews (12), surveys (8), FGs and interviews (1), FGs (5), observational (1) | 4/9 | - Perceptions of the knowledge or skills needed to support behaviour change with patients (barrier) - Perceived lack of time to deliver behaviour change interventions - Contextual enablers - Beliefs about resources and support needed to facilitate intervention delivery (barrier) - Beliefs about resources and support needed to facilitate intervention delivery (enabler) - Healthcare professionals’ perceptions of patient motivation - Training as an enabler to delivering behaviour change interventions - Attitudes towards delivering behaviour change interventions - Perceptions of the healthcare professional role (enabler) |
| Yousefzadeh 2016 | Narrative using systematic search | Not stated | Not stated | Anaesthesiologists | 95 | Not stated | 2/9 | - Perceived lack of time to deliver behaviour change interventions - Contextual enablers - Perceptions of the knowledge or skills needed to support behaviour change with patients (barrier) |
| Zhu 2011 | Systematic review | Quantitative | Not stated | Registered nurses (3), GPs (3), gamily physicians (3), school nurses (1), paediatricians (2), registered dieticians (1), paediatric nurse practitioners (1) | 12 (11 including a hcp) | Cross-sectional survey (10), quasi-experimental post-test design (1) | 4/9 | - Healthcare professionals’ own health behaviour (barrier) - Perceptions of the knowledge or skills needed to support behaviour change with patients (barrier) |
